# Evidence of Polygenic Adaptation in the Systems Genetics of Anthropometric Traits

**DOI:** 10.1371/journal.pone.0160654

**Published:** 2016-08-18

**Authors:** Renato Polimanti, Bao Zhu Yang, Hongyu Zhao, Joel Gelernter

**Affiliations:** 1 Department of Psychiatry, Yale University School of Medicine, West Haven, Connecticut, United States of America; 2 VA CT Healthcare Center, West Haven, Connecticut, United States of America; 3 Department of Biostatistics, Yale University School of Public Health, New Haven, Connecticut, United States of America; 4 Department of Genetics, Yale University School of Medicine, New Haven, Connecticut, United States of America; 5 Department of Neurobiology, Yale University School of Medicine, New Haven, Connecticut, United States of America; University of Florence, ITALY

## Abstract

Many signals of natural selection have been identified in the human genome. However, except for some single-locus mechanisms, most molecular processes generating these adaptation signals are still unknown. We developed an approach that integrates datasets related to genome-wide association studies (GWAS) with information about systems biology and genetic signatures of natural selection to identify evidence of polygenic adaptation. Specifically, we focused on five anthropometric measurements: body mass index (BMI), height, waist-to-hip ratio adjusted for BMI (WHR), and waist circumference adjusted for BMI (WC), and sex differences for WHR and WC. We performed an enrichment analysis for signals of natural selection in protein interaction networks associated with anthropometric traits in European populations. The adaptation signals-enriched gene networks associated highlighted epistatic interactions in the context of polygenic selection for the investigated traits. These polygenic mechanisms indicated intriguing selective mechanisms related to the anthropometric traits: adult locomotory behavior for BMI, infection resistance for height, interplay between lipid transport and immune systems for WHR, and female-specific polygenic adaptation for WHR and WC. In conclusion, we observed evidence of polygenic adaptation in the context of systems genetics of anthropometric traits that indicates polygenic mechanisms related to the natural selection in European populations.

## Introduction

Numerous studies have investigated the human genome to understand how human species adapted to the very different environments worldwide [1, 2]. The explosion of high-throughput technologies and informatics permitted development of methods based on genome-wide data to detect signatures of natural selection in the human genome [3]. Accordingly, reliable evidence of genetic signals of selection across the genome has been identified regarding human adaptations to infections [4], ultraviolet radiation [5], diet [6], and high altitude [7]. However, some investigators have argued that the identified single-locus signals of selection represent only a small part of the process of shaping adaptation-related human genomic variation [8, 9]; and that most adaptation events in natural populations occur via polygenic mechanisms, in accordance with quantitative genetics models. Genome-wide association studies (GWAS) have widely demonstrated that complex traits are substantially attributable to many independent loci with small effect sizes [10] and genetic adaptation can affect GWAS outcomes [11]. A broader view of these findings in the context of natural selection is most consistent with the view that selection of phenotypic traits in response to environmental pressures probably generally occurs via polygenic adaptation, rather than sudden large-effect mutations at individual key loci. Although these data strongly encourage exploration of polygenic mechanisms, this is still a field in its infancy. In 2012, Turchin and colleagues detected signature of widespread selection in height-associated loci [12]. Specifically, they observed that frequencies of alleles associated with increased height are systematically elevated in Northern Europeans compared with Southern Europeans. In 2013, Daub and colleagues used a different approach based on F_ST_ probabilities, pathway-enrichment analysis, and long-distance genotypic linkage disequilibrium (LD) to investigate polygenic adaptation in human genome [13]. They showed that pathogen-human interactions have produced widespread and coordinated genomic responses, indicating that adaptation to pathogens is a good example of polygenic selection. Berg and Coop investigated polygenic adaptation among the loci associated with different complex traits (i.e., height, skin pigmentation, body mass index (BMI), type 2 diabetes, Crohn’s disease, and ulcerative colitis) by GWAS; they observed a number of putative signals of local adaptation [14]. These investigations provided evidence regarding polygenic adaptation and methods useful to investigate polygenic adaptation.

Compared to the previous studies which used information about GWAS significant outcomes or pre-defined molecular pathways, we employed GWAS datasets (i.e., data about millions of variants across the human genome) together with information about systems biology and genetic signatures of natural selection. To test our approach, we focused this investigation on different sub-phenotypes (i.e., distribution, phenotypic variability, extreme phenotype differences, and sex-differences) related different anthropometric traits (BMI, height, waist-to-hip ratio adjusted for BMI (WHR), and waist circumference adjusted for BMI (WC)), since anthropometric traits have been affected by numerous different environmental pressures in the process of human evolution and adaptation, including known adaptation mechanisms that link anthropometric traits with climate [15], diet [16], and fertility [17]. Furthermore, since the polygenic inheritance of anthropometric traits seems not attributable to lower-frequency variants [18], these traits are good candidates to be investigated by our approach that focused attention on polygenic inheritance from common variants. In our investigation, we performed iHS (integrated Haplotype score) enrichment analysis of protein interaction networks associated with anthropometric traits, tested the epistatic interactions among loci included in the iHS-enriched protein networks, and verified the enrichments of the interactive loci for gene ontologies and known molecular pathways. Since haplotype structure-based statistics can identify selective sweeps under a number of different selection scenarios (i.e., complete, incomplete hard, and soft sweeps) [19], our iHS-based strategy is capable of detecting polygenic adaptation related to several different selection mechanisms. Our findings indicated evidence of polygenic adaptation in the context of systems genetics of anthropometric traits that are related to different biological mechanisms.

## Materials and Methods

### GWAS summary statistics

We considered the large-scale meta-analysis of GWAS performed by GIANT (Genetic Investigation of ANthropometric Traits) consortium for BMI [20–22], height [21–23], WHR [22, 24, 25], and WC [25]. GIANT data were downloaded from http://www.broadinstitute.org/collaboration/giant/index.php/GIANT_consortium_data_files in August 2014. For each anthropometric trait, we investigated GWAS data related to the analyses based on different sub-phenotypes: distribution, phenotypic variability, and extreme phenotype differences (S1 Table). The GIANT investigators observed sex differences in the genetics of WC and WHR [25], so we analyzed male- and female-specific GWAS data for these two traits separately. Regarding WC and WHR analysis, we focused on BMI-adjusted traits since these adjusted traits provide information about body shape and fat accumulation distribution. Since no summary statistics are publically available about large GWAS of anthropometric traits in non-European populations, we restricted our analysis to the GIANT data and the genetic variation within European ancestry. Our recent study demonstrated that GWAS findings depends to the ancestry genomic background [11]. Accordingly, summary statistics of GWAS performed on European populations cannot be used to investigate other human groups.

### Integrated haplotype score screening in protein interaction networks

We used information about iHS–a statistic based on the differential levels of LD able to detect evidence of recent positive selection [26]–to focus our analysis on loci which are known to have suggestive signals of natural selection. We chose this specific selection metric because this method is appropriate to detect non-fixed selected alleles, like those expected in a polygenic adaptation scenario [9], in a specific population. A recent study confirmed that haplotype structure-based statistics can identify selective sweeps under several different selection scenarios (i.e., complete, incomplete hard, and soft sweeps) [19].

Since GIANT performed their GWAS on European ancestry populations, we used the iHS data based on the HapMap Phase 2 CEU (Utah residents with Northern and Western European ancestry from the CEPH collection) population available in Haplotter database [26]. Previous studies used |iHS| > 1.5 as suggestive evidence for natural selection [27–29], in agreement with a recent simulation analysis that indicated |iHS| > 2.0 is too stringent a threshold [30]. Accordingly, we selected from GIANT GWAS datasets those variants with |iHS| > 1.5 (S1 Table). Considering iHS selection criterion, we performed a gene-based association analysis using VEGAS software [31]. The HapMap CEU reference panel was used to correct for LD patterns. We used the summary statistics of gene-based association analysis to perform an association analysis based on protein-protein interactions (PPI) using the R package dmGWAS [32]. We defined PPIs of all genes with gene-based association using the Protein Interaction Network Analysis (PINA) platform v2.0 [33] that includes information about physical and genetic PPIs. For each investigated trait, we further considered analysis for the associated gene network defined by the top-10 PPI modules (S2 Table). We developed this multi-step approach based on iHS screening and gene- and PPI-based analyses to apply the iHS enrichment analysis on protein interaction networks, instead of a continuous genome region. This approach can improve the effectiveness of iHS screening to detect polygenic adaptation rather than single-locus signals.

### Long-distance genotypic Linkage Disequilibrium

As previously performed by Daub and colleagues [13], we used long-distance genotypic LD to detect epistatic interactions among variants included in the protein networks enriched for selection signals. For each trait, we calculated the long-distance genotypic LD among the variants located in the genes included in the top-10 PPI modules using Genepop 4.2 software [34]. To reduce the number of variants carried forward to investigate in the next steps, we pruned the variants for LD (r^2^ > 0.8) using PLINK software [35]. Both data pruning and genotypic LD analysis were performed using HapMap Phase 3 CEU population [36]. Long-distance LD pairs were defined as pairs of variants either (a) mapped to different chromosomes, or (b) on the same chromosome separated by ≥ 10 Mb. Furthermore, we excluded LD pairs where none of the variants is associated with the anthropometric trait at a nominal significance level. We used the R package “qvalue” to estimate q-values for controlling the false discovery rate (FDR), and considered q-values < 0.05 as significant.

### Term Enrichment analysis

Considering the genes with significant evidence of epistatic interactions (long-distance genotypic LD with FDR q<0.05), we generated a gene set for each investigated anthropometric trait potentially and analyzed the enrichment for different categorical data (e.g., gene ontology and molecular pathway membership) using DAVID 6.7 software [37].

## Results

### Body Mass Index

**Fig 1:**
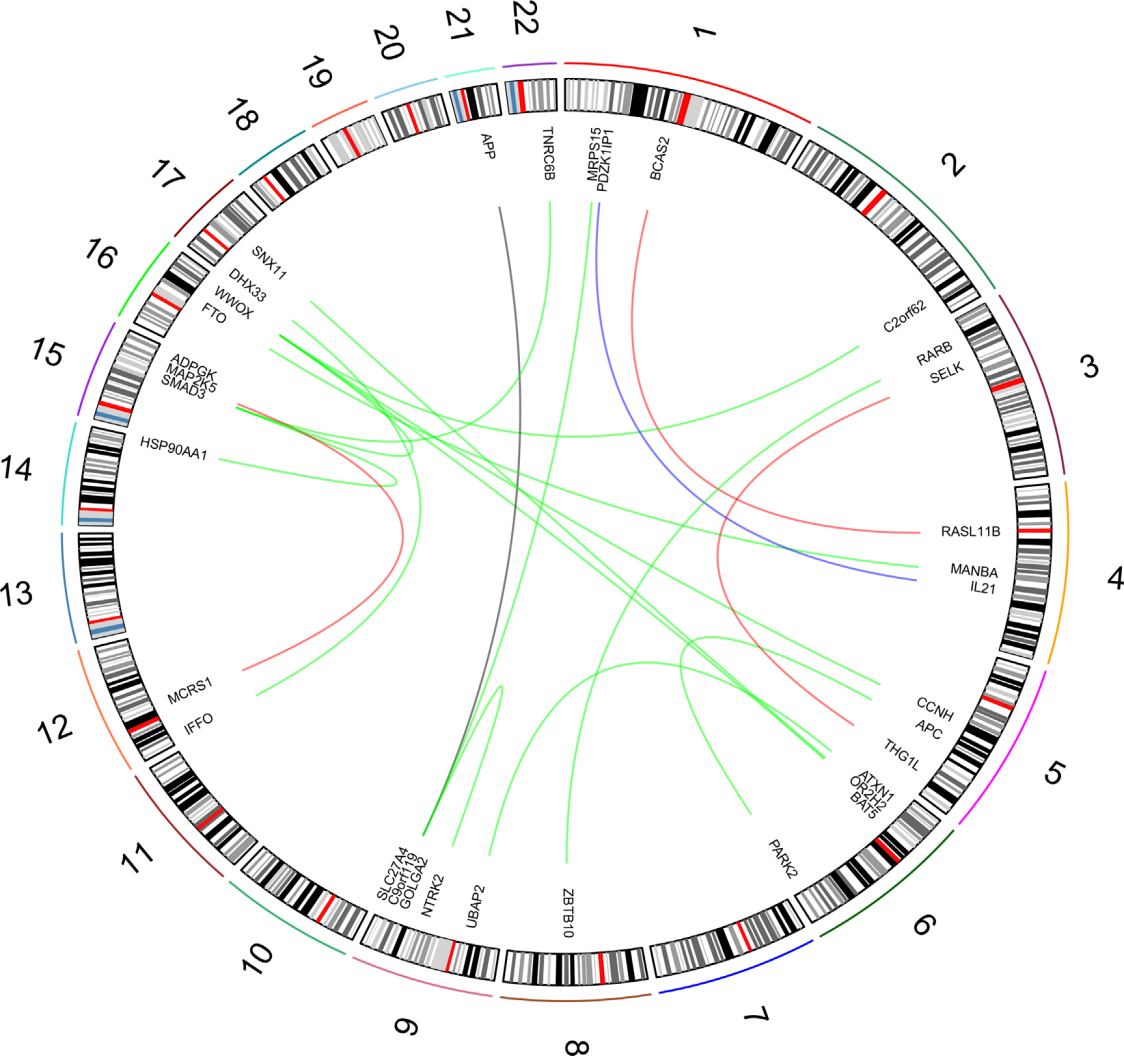
Significant long-distance genotypic LD in BMI-associated gene network. The BMI phenotypic analyses are highlighted with different colors: green (distribution), red (phenotypic variability), blue (extreme phenotypes), and black (overlapping between distribution and extreme-phenotype analyses).

**Fig 2:**
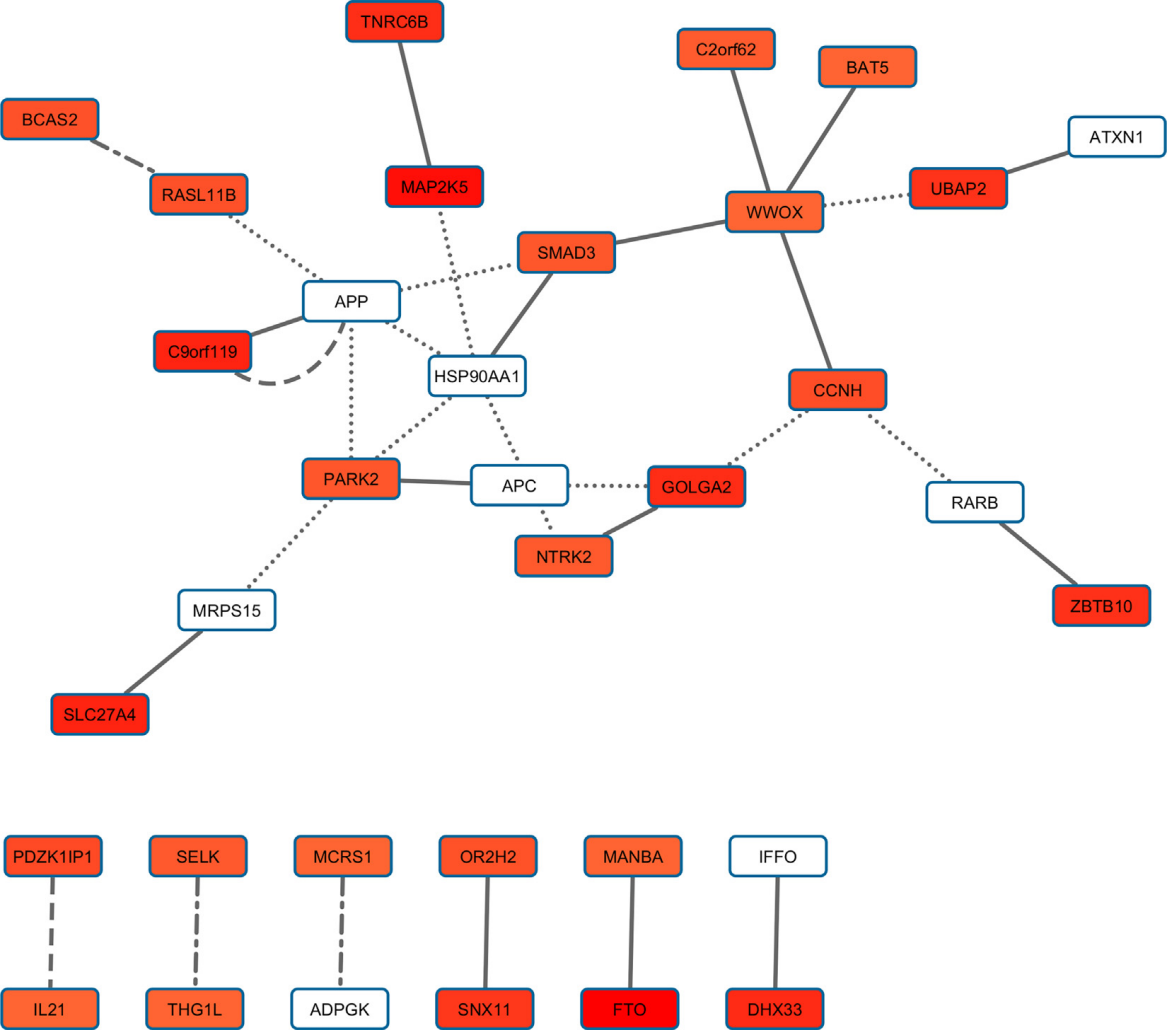
Gene set with evidences of polygenic adaptation in the systems genetics of BMI-related sub-phenotypes. The intensity of the box shadings is proportional to the strength of the gene-based significant association. The types of line indicate the source of interaction evidence: solid line (BMI distribution analysis), dashed line (BMI extreme phenotype analysis), dotdash line (phenotypic variability analysis), and dotted line (PINA database).

BMI PPI-based analysis generated three gene networks associated with the investigated BMI-related sub-phenotypes (i.e., distribution, phenotypic variability, and extreme phenotype differences). The three BMI-related gene networks (S3 Table) respectively include 201 genes (BMI distribution; 1,013 pruned variants with |iHS| > 1.5), 93 genes (BMI phenotypic variability; 395 pruned variants with |iHS| > 1.5), and 79 genes (BMI extreme phenotype differences; 332 pruned variants with |iHS| > 1.5). Calculating the long-distance genotypic LD, we observed significant LD pairs (Fig 1; S4 Table): 20 in the BMI distribution gene network, three in the BMI phenotypic-variability gene network, and two in the BMI extreme-phenotypic-difference gene network. Considering the information about long-distance genotypic LD and PPIs, we generated a BMI gene set based on the putative signals of polygenic adaptation in the systems genetics of BMI (Fig 2). The enrichment analysis in this gene set indicated that it is significantly associated with adult locomotory behavior, learning, and various molecular regulatory mechanisms (Table 1).

**Table 1:** Term enrichment analysis of gene sets with evidences of polygenic adaptation. Fisher exact test p values adjusted for Bonferroni correction are reported.

Anthropometric trait	Sub-phenotype	Term	Genes	adjusted P value
BMI	distribution	GO:0008344~adult locomotory behavior	*ATXN1*, *APP*, *PARK2*	1.64E-02
		GO:0007612~learning	*ATXN1*, *APP*, *PARK2*	2.28E-02
		GO:0050678~regulation of epithelial cell proliferation	*SMAD3*, *MAP2K5*, *APC*	3.80E-02
		GO:0045944~positive regulation of transcription from RNA polymerase II promoter	*ATXN1*, *APP*, *CCNH*, *SMAD3*, *RARB*	4.30E-02
	extreme phenotype differences	GO:0048469~cell maturation	*APP*, *IL21*	6.66E-04
		GO:0021700~developmental maturation	*APP*, *IL21*	1.48E-03
Height	Distribution	OMIN—Genomewide Association Study of an AIDS-Nonprogression Cohort Emphasizes the Role Played by HLA Genes	*MICB*, *RNF39*, *HCP5*, *HLA-B*	7.32E-04
		OMIN—Genome-wide association analysis identifies 20 loci that influence adult height	*DYM*, *ACAN*, *CDK6*, *SCMH1*	3.22E-02
		GO:0046703~natural killer cell lectin-like receptor binding	*MICB*, *MICA*	3.90E-02
	Extreme phenotype differences	GO:0030235~nitric-oxide synthase regulator activity	*HSP90AA1*, *ESR1*	2.39E-02
		GO:0042802~identical protein binding	*ATXN1*, *APP*, *HSP90AA1*, *ATG7*, *SMAD3*, *GNAS*, *TTN*, *CDSN*	4.86E-02
WHR	Men	GO:0044431~Golgi apparatus part	*CORO7*, *SNAP25*	7.48E-03
		GO:0005624~membrane fraction	*CORO7*, *SNAP25*	1.87E-02
		GO:0005626~insoluble fraction	*CORO7*, *SNAP25*	2.02E-02
		GO:0000267~cell fraction	*CORO7*, *SNAP25*	2.64E-02
	Women	GO:0006955~immune response	*IKBKAP*, *APOL1*, *PPARG*, *HLA-B*	1.28E-03
		GO:0006952~defense response	*APOL1*, *PPARG*, *HLA-B*	3.48E-02
		GO:0045087~innate immune response	*APOL1*, *PPARG*	4.00E-02
		GO:0006869~lipid transport	*APOL1*, *PPARG*	4.40E-02

### Height

**Fig 3:**
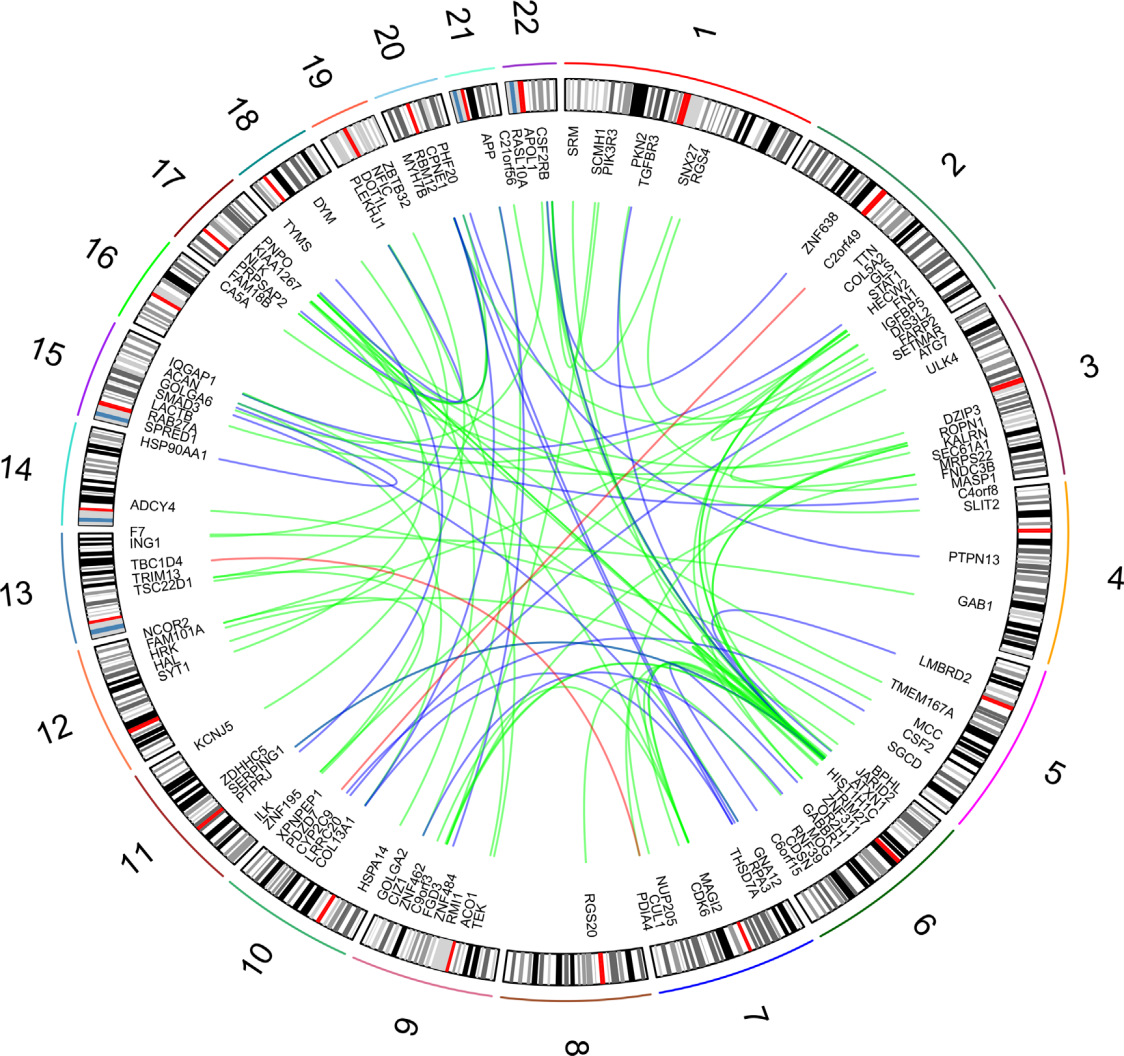
Significant long-distance genotypic LD in Height-associated gene network. The height phenotypic analyses are highlighted with different colors: green (distribution), red (phenotypic variability), and blue (extreme phenotypes).

**Fig 4:**
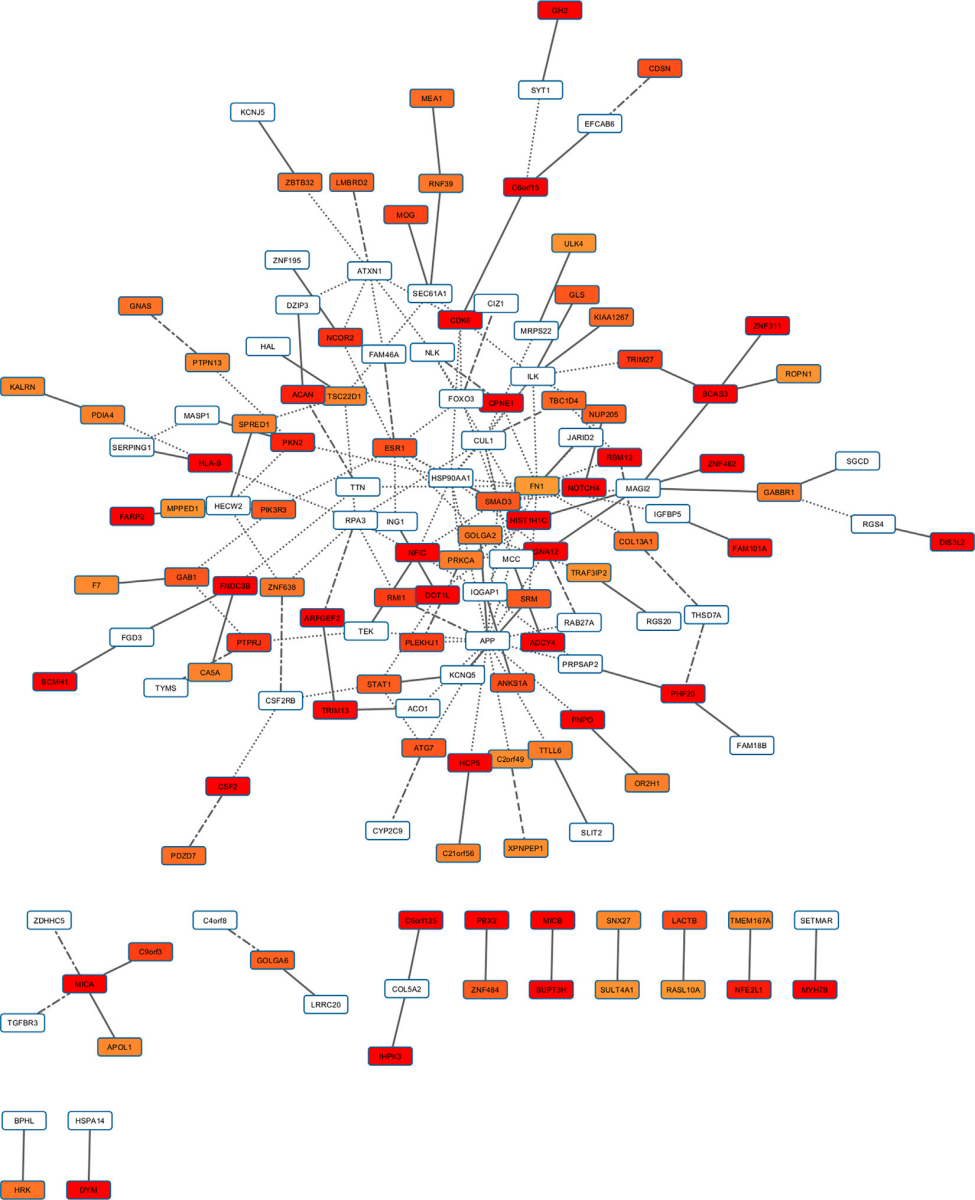
Gene set with evidences of polygenic adaptation in the systems genetics of Height-related sub-phenotypes. The intensity of the box shadings is proportional to the strength of the gene-based significant association. The types of line indicate the source of interaction evidence: solid line (height distribution analysis), dashed line (height phenotypic variability analysis), dotdash line (height extreme phenotype analysis), and dotted line (PINA).

Height PPI-based association analysis produced three gene networks for the investigated height-related sub-phenotypes (i.e., distribution, phenotypic variability, and extreme phenotype differences). Specifically, the height-related gene networks (S5 Table) respectively include 532 genes for height distribution (2,246 pruned variants with |iHS| > 1.5), 114 genes for phenotypic variability (447 pruned variants with |iHS| > 1.5), and 409 genes for extreme phenotype differences (1,692 pruned variants with |iHS| > 1.5). The analysis of long-distance genotypic LD identified numerous significant LD pairs (Fig 3; S6 Table): 68 in the height distribution gene network, two in the height phenotypic-variability gene network, and 25 in the height extreme-phenotypic-difference gene network. The gene sets made using the information about the long-distance genotypic LD and PPIs suggested numerous interacting processes in polygenic adaptation related to height (Fig 4). The enrichment analysis indicated that this height-related gene set is significantly associated with GWAS outcomes of AIDS-nonprogression phenotype, GWAS results for height, immune systems-related functions, and other molecular processes (Table 1).

### Waist circumference

**Fig 5:**
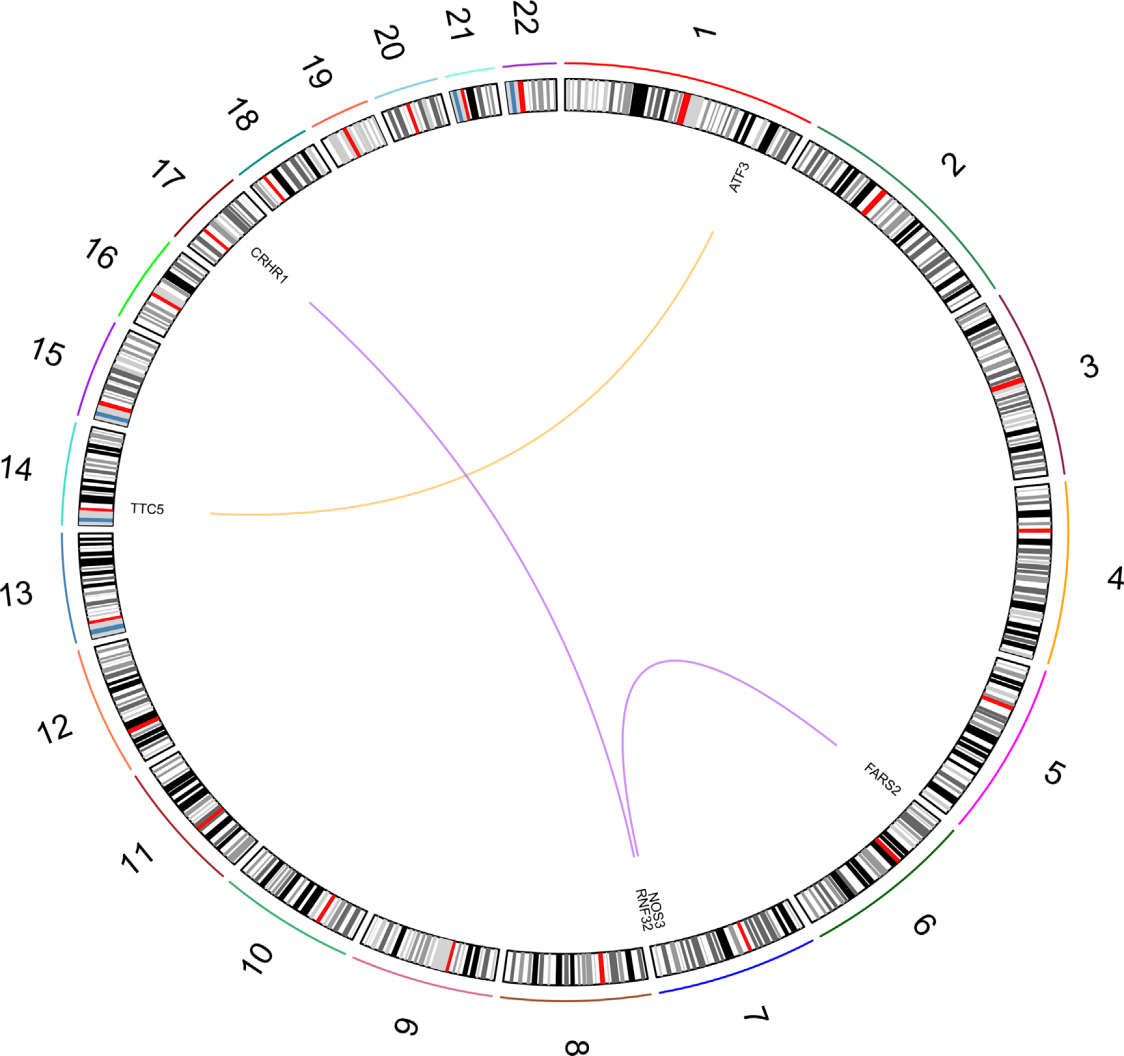
Significant long-distance genotypic LD in WC-associated gene network. The WC phenotypic analyses are highlighted with different colors: yellow (men) and purple (women).

**Fig 6:**
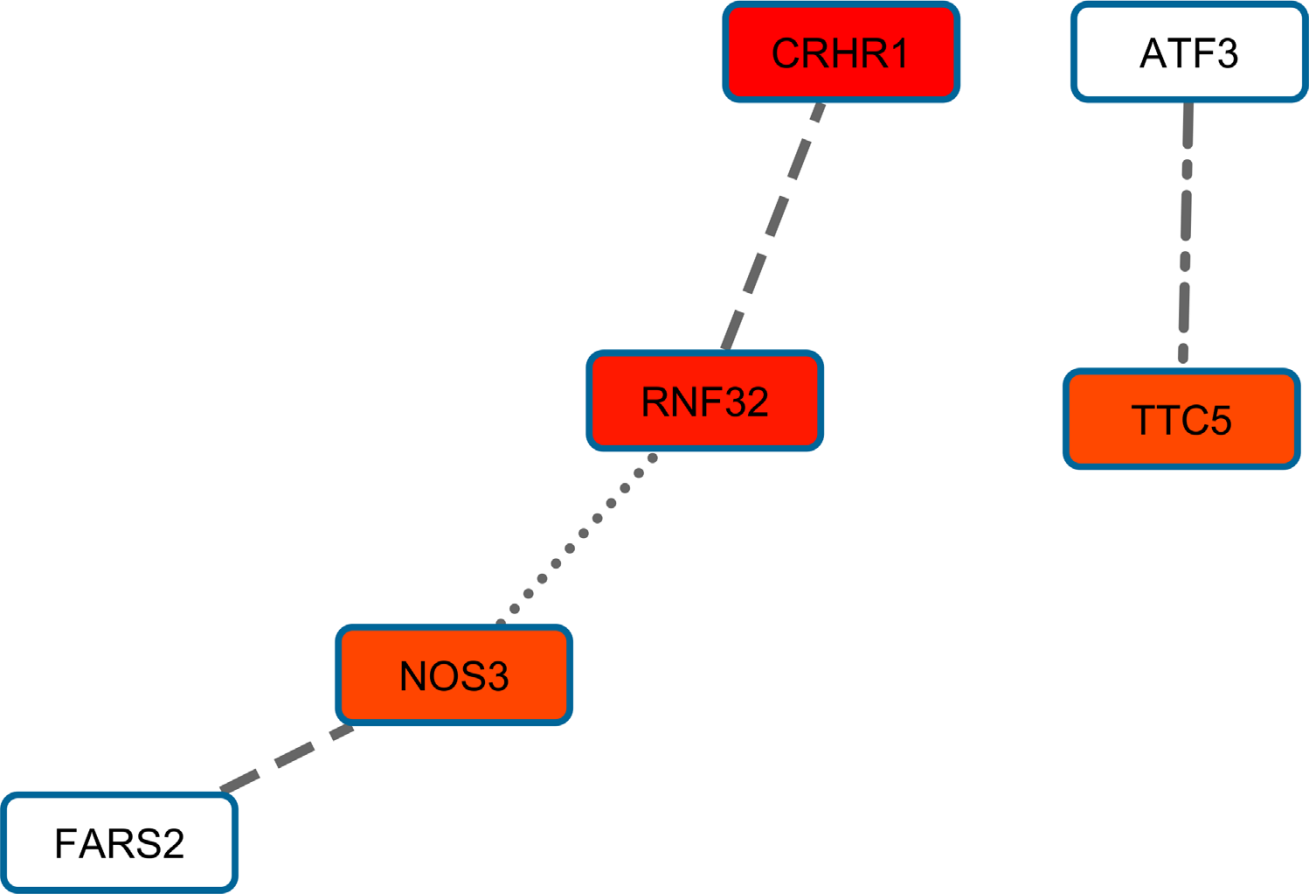
Gene set with evidences of polygenic adaptation in the systems genetics of WC-related sub-phenotypes. The intensity of the box shadings is proportional to the strength of the gene-based significant association. The types of line indicate the source of interaction evidence: dashed line (women WC analysis) and dotted line (men WC analysis).

For WC, GWAS datasets were available only for sex differences (i.e., men and women). WC PPI-based association analysis generated a gene network for each investigated WC-related sub-phenotype (S7 Table). A total of 148 genes were included in the male WC gene network (715 pruned variants with |iHS| > 1.5), and 77 genes in the female WC gene network (328 pruned variants with |iHS| > 1.5). In these gene networks, we observed one and two significant long-distance genotypic LD pairs, respectively (Fig 5; S8 Table). Fig 6 reports the WC gene set based on genotypic LD analysis and PPI information. No significant enrichments were observed in the WC gene set.

### Waist-to-hip ratio

**Fig 7:**
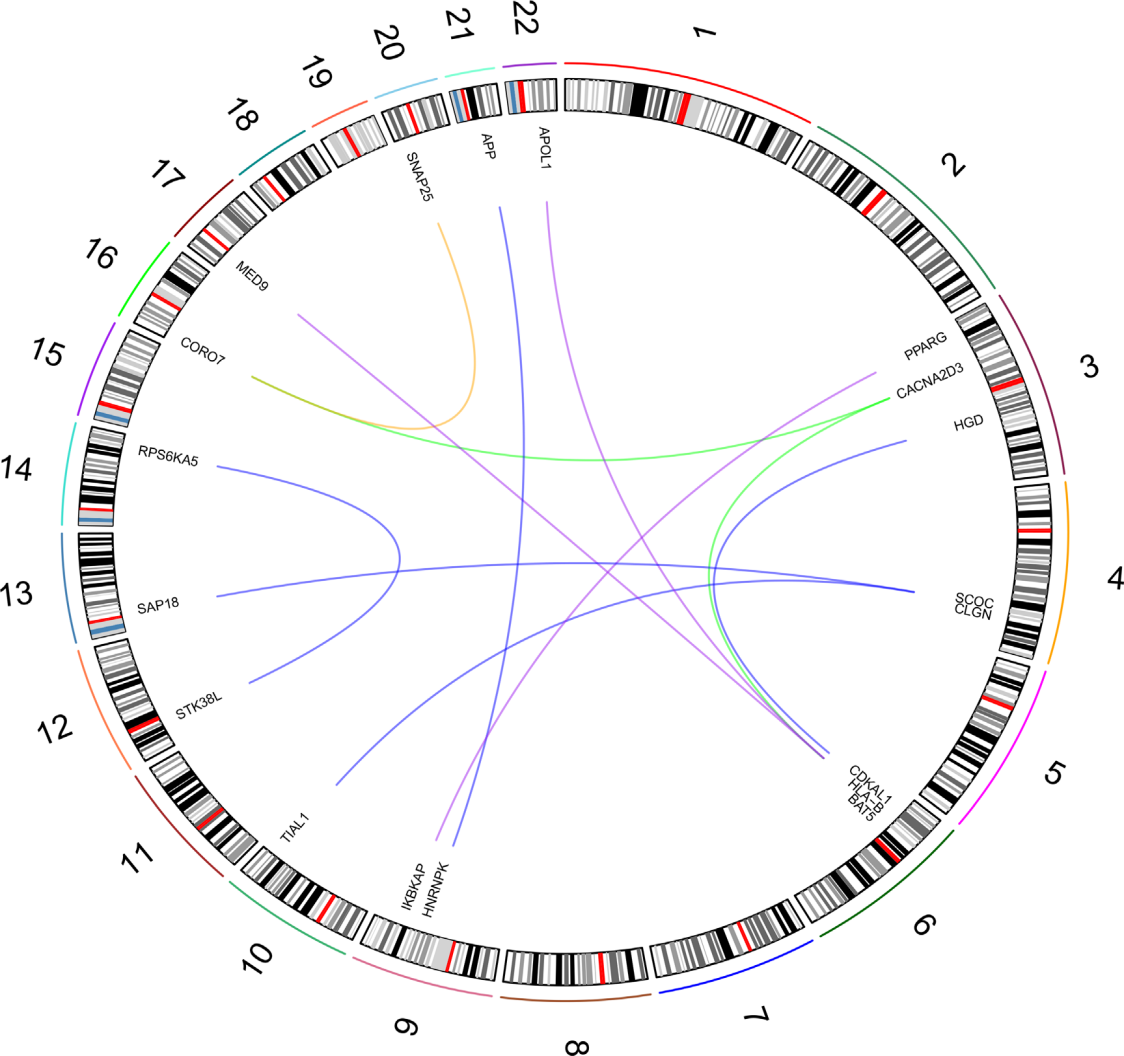
Gene set with evidences of polygenic adaptation in the systems genetics of WHR-related sub-phenotypes. The intensity of the box shadings is proportional to the strength of the gene-based significant association. The types of line indicate the source of interaction evidence: solid line (WHR distribution analysis), dashed line (WHR extreme phenotype analysis), dotdash line (WHR men analysis), double line (WHR women analysis), and dotted line (PINA).

**Fig 8:**
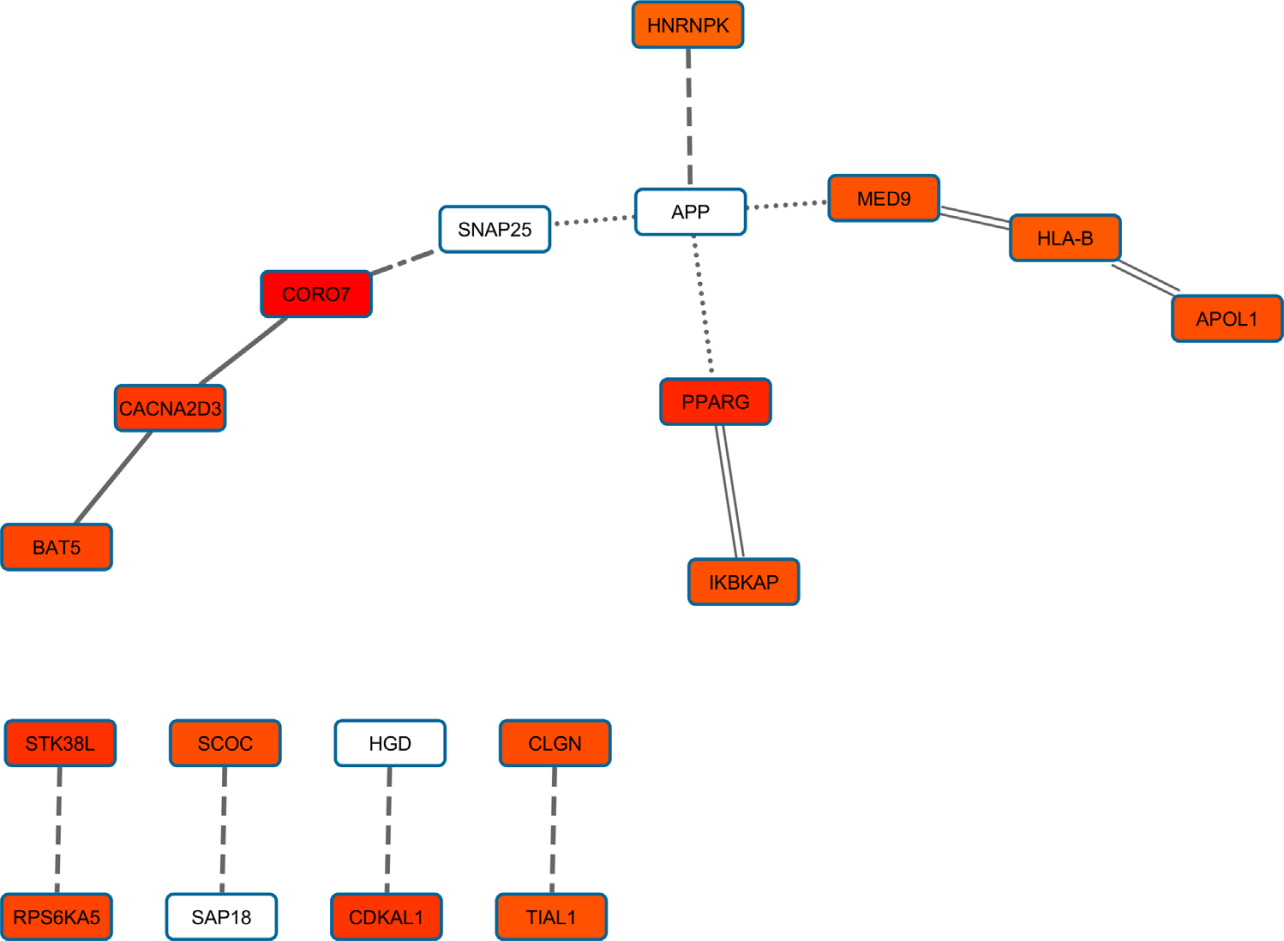
Significant long-distance genotypic LD in WHR-associated gene network. The WHR phenotypic analyses are highlighted with different colors: green (distribution), blue (extreme phenotypes), yellow (men) and purple (women).

In WHR analysis, we considered four sub-phenotypes: distribution and extreme phenotype differences, and males and females. PPI-based association analysis defined a gene network for each investigated WHR sub-phenotype (S9 Table). One hundred thirty-nine genes are included in the WHR-distribution gene network (702 pruned variants with |iHS| > 1.5), 138 in the WHR-extreme-phenotype-difference gene network (578 pruned variants with |iHS| > 1.5), 65 genes in the WHR-men gene network (257 pruned variants with |iHS| > 1.5), and 92 genes in the WHR-women gene network (406 pruned variants with |iHS| > 1.5). Among these WHR-related gene networks, we observed significant long-distance genotypic LD (Fig 7; S10 Table): two significant pairs in WHR distribution, five significant pairs in WHR-extreme-phenotype-difference, one significant pair in WHR-males, and four significant pairs in WHR-females. Fig 8 reports the WHR gene sets based on long-distance genotypic LD and PPIs. The enrichment analysis indicates a significant association for cellular structure-related terms in male WHR gene sets, and terms related to immune systems and lipid transportation in female WHR gene sets (Table 1).

## Discussion

Our results highlight evidences of polygenic adaptation in the systems genetics of anthropometric traits, suggesting that polygenic adaptation is not uncommon in the systems genetics of complex traits, and confirming that anthropometric traits are influenced by several selective pressures related to biological mechanisms, such as behaviors and immune systems. Our approach demonstrated the usefulness of combining information from GWAS and systems biology in investigating polygenic mechanisms in human adaptation. Previous studies of polygenic adaptation obtained interesting results [12–14], focusing on main genetic risk factors or specific pathways, and investigating other natural selection signatures in human genome (e.g., F_ST_). In our study, we applied a different approach based on GWAS summary statistics, gene interactive network and iHS. Since polygenic mechanisms mostly involve multiple loci with small effects that are implicated in different molecular pathways [10], our approach appears, at least in these instances, to be effective in detecting polygenic selection in the systems genetics of complex traits. Thus we have demonstrated utility in combining GWAS of complex traits with information about systems biology and natural selection signatures. Furthermore, we also confirmed the recent analysis of Ferrer-Admetlla and colleagues [19] regarding haplotype structure-based statistics since our iHS-based analysis was able to detect the widespread signals of polygenic adaptation.

In the BMI analysis, an examination of the systems genetics of this trait resulted in a conclusion that there is evidence of polygenic adaptation in the systems genetics of this trait. Most significant epistatic interactions (i.e., significant long-distance genotypic LD) among variants with suggestive signatures of natural selection (i.e., |iHS| > 1.5) are related to the analysis of BMI distributions. This is almost certainly due to the fact that GWAS of BMI distribution identified more loci associated with this sub-phenotype than the other BMI-related sub-phenotypes, and therefore this comparison had better power. The enrichment analysis of the BMI gene sets related to polygenic adaptation indicated significant association with some gene ontologies. Among them, the most obviously relevant is related to adult locomotory behavior and learning. Adult locomotory behavior is defined as the specific movement from place to place of a fully developed and mature organism in response to external or internal stimuli. Animal experiments explored different molecular aspects involved in stimulus-response activities, highlighting the interplay between food intake and locomotory behavior to maintain energy homoeostasis [38–40]. No previous study highlighted putative signals of natural selection in relation to genes involved in these mechanisms. Our data support an intriguing scenario where genes involved in adult locomotory behavior are under selective pressure, and this could affect the systems genetics of BMI. Furthermore, recent data indicated that some BMI-associated loci mechanisms may be present in specific population categories [41], suggesting additional pathogenic mechanisms that differ from those present in the general population.

In the height analysis, we identified a large gene set with evidence of polygenic adaptation. This is consistent with both the previous studies regarding natural selection related to height [12, 14] and the large number of genome-wide significant loci identified by GWAS of height [23, 42]. Our results provide additional information about the interplay between height and immune system. The gene set with evidence of polygenic adaptation related to height distribution is enriched in genes identified by GWAS of AIDS resistance [43], and genes encoding natural killer cell lectin-like receptor binding. Several studies demonstrated the relevant relationship between infection occurrences and stature, suggesting a relevant interplay between nutrition and infection [43, 44]. Furthermore, a recent study indicated high frequency of short stature in HIV-infected children, and poor adherence to antiretroviral treatment, severe immunosuppression, and therapy inefficacy are associated with severe short stature.[45] Accordingly, it is a reasonable hypothesis that greater infection susceptibility is one reason that individuals might fail to attain what would otherwise be their genetically-determined greatest height; and that adaptation processes related to infection resistance could also play a role in the genetics of height. Moreover, one of the previous studies about polygenic adaptation found widespread and coordinated genomic responses related to the adaptation to pathogens [13], consistent with our findings about polygenic selection in immune system-related genes.

WC analysis identified little evidence of polygenic adaptation, and what was observed was mainly in females. No significant term enrichments were observed. However, among the genes included in the female WC gene set, three may be interesting: *CRHR1*, *NOS3*, and *FARS2*. *CRHR1* is a genome-wide significant locus for bone-mineral-density [46] and a suggestive locus for infant head circumference [47], and it is also involved in stress, reproduction, immune response, and obesity [48, 49]. *NOS3* is involved in several sex-specific molecular processes, including cardiac and endothelial function [50, 51]. *FARS2* is a suggestive locus (p<10^−7^) for severe early-onset obesity [52].

The investigation of WHR data indicated putative signals of polygenetic adaptation in the systems genetics of this trait. In particular, the female-specific findings suggest interplay between lipid metabolism and immune systems in the enrichment analysis. The most immediately relevant locus involved in this gene set with evidence polygenic adaptation is *PPARG*. This gene was genome-wide significant in a GWAS of WHR in women [25]. Furthermore, population genetics studies indicated that it is a candidate in relation to metabolic adaptation [53]. Finally, investigations using different approaches uncovered several functions of this gene and its involvement in the pathogenesis of obesity, diabetes, atherosclerosis, and cancer [54]. The enrichment analysis based on the *PPARG* gene and the other genes involved in polygenic adaptation related to female WHR (i.e., *IKBKAP*, *APOL1*, *HLA-B*, and *MED9*) indicated an overlapping between immune systems and lipid transportation. Both these molecular processes are widely recognized as mechanisms under selective pressures during human evolution [13, 55], and molecular investigations have also highlighted the strong interplay between them [56, 57]. Furthermore, the sexes showed significant differences in both these mechanisms, mainly attributable to the female reproductive function [58, 59]. All these results are consistent with the possibility of polygenic adaption observed in the female WHR gene set.

In conclusion, our study suggests an effective approach to the investigation of polygenic adaption in the systems genetic of complex traits based on combining GWAS data with information regarding the systems genetics of complex traits. Regarding the results obtained about anthropometric traits, our findings lead to novel insights that indicate polygenic adaptation in response to selective pressures related to locomotory behavior, infection resistance, and lipid transport. Further, in sex-stratified analysis of WC and WHR, we observed most of the significant findings in females. These data indicate that the effect of past adaptation processes (e.g., locomotory behaviors and infection resistance) on human genome variation are affecting the predisposition to anthropometric traits. However, these findings are specific to the genetic structure of CEU populations that is the most used reference panel for European populations. Additional polygenic adaptation processes are expected for other European and non-European populations.

Besides its potential contribution to population genetics, our study also suggested the understanding of epistatic interactions related to polygenic adaptation may also improve our knowledge about systems genetics, highlighting novel putative interactive processes to investigate. Finally, here we focused on anthropometric traits, obtaining interesting results about the potential role of human adaptation in shaping the genetic predisposition to these traits. However, numerous GWAS complex traits datasets are available to be investigated. The exploration of these data may provide further information useful to understand both the genetic mechanisms of human adaptation, and their effects on the genetic predisposition to complex traits.

## Supporting Information

S1 Table GWAS data of anthropometric traits made available by GIANT consortium that were used in the present study. (DOCX)

S2 Table gene networks associated with GIANT phenotypic traits that were constructed on the basis of top-10 PPI modules constructed with variants with |iHS| > 1.5. (DOCX)

S3 Table Genes and their correspondence p values present in gene network associated with BMI-related phenotypes. NI: not included in top-10 PPI modules.
(DOCX)

S4 Table Significant long-distance genotypic LDs observed in BMI-associated gene networks. (DOCX)

S5 Table Genes and their correspondence p values present in gene network associated with height-related phenotypes. NI: not included in top-10 PPI modules.
(DOCX)

S6 Table Significant long-distance genotypic LDs observed in Height-associated gene networks. (DOCX)

S7 Table Genes and their correspondence p values present in gene network associated with WC-related phenotypes. NI: not included in top-10 PPI modules.
(DOCX)

S8 Table Significant long-distance genotypic LDs observed in WC-associated gene networks. (DOCX)

S9 Table Genes and their correspondence p values present in gene network associated with WHR-related phenotypes. NI: not included in top-10 PPI modules.
(DOCX)

S10 Table Significant long-distance genotypic LDs observed in WHR-associated gene networks. (DOCX)
