# Supplementary material for: Evidence of Polygenic Adaptation in the Systems Genetics of Anthropometric Traits
Source: PLoS One. 2016 Aug 18;11(8):e0160654. doi: 10.1371/journal.pone.0160654 (PMC4990182; doi:10.1371/journal.pone.0160654)
Supplement: S1 Table — (DOCX) [file pone.0160654.s001.docx]

**S1 Table**: GWAS data of anthropometric traits made available by GIANT consortium that were used in the present study.

| **Anthropometric trait** | **Phenotype** | **Total Variants** | **Variants with \|iHS\|** **> 1.5** | **References** |
| --- | --- | --- | --- | --- |
| BMI | Distribution | 2,471,516 | 278,547 | Speliotes et al., 2010 Nat Genet |
|  | Phenotypic variability | 2,417,848 | 277,443 | Yang et al., 2012 Nature |
|  | Extreme phenotype differences | 1,984,813 | 253,638 | Berndt et al., 2013 Nat Genet |
| Height | Distribution | 2,469,635 | 278,463 | Lango Allen et al., 2010 Nature |
|  | Phenotypic variability | 2,417,848 | 277,443 | Yang et al., 2012 Nature |
|  | Extreme phenotype differences | 1,966,556 | 251,577 | Berndt et al., 2013 Nat Genet |
| WC | Men | 2,744,339 | 279,526 | Randall et al., 2013 Plos Genet |
|  | Women | 2,738,311 | 279,529 | Randall et al., 2013 Plos Genet |
| WHR | Distribution | 2,483,325 | 282,982 | Heid et al., 2010 Nat Genet |
|  | Extreme phenotype differences | 1,939,900 | 248,632 | Berndt et al., 2013 Nat Genet |
|  | Men | 2,742,718 | 279,524 | Randall et al., 2013 Plos Genet |
|  | Women | 2,737,329 | 279,531 | Randall et al., 2013 Plos Genet |
