# Supplementary material for: Evidence of Polygenic Adaptation in the Systems Genetics of Anthropometric Traits
Source: PLoS One. 2016 Aug 18;11(8):e0160654. doi: 10.1371/journal.pone.0160654 (PMC4990182; doi:10.1371/journal.pone.0160654)
Supplement: S2 Table — (DOCX) [file pone.0160654.s002.docx]

**S2 Table**: gene networks associated with GIANT phenotypic traits that were constructed on the basis of top-10 PPI modules constructed with variants with |iHS| > 1.5.

| **Anthropometric trait** | **Phenotype** | **Gene N** | **variant N** | **pruned variant N** |
| --- | --- | --- | --- | --- |
| BMI | Distribution | 201 | 1,961 | 1,013 |
|  | Phenotypic variability | 93 | 752 | 395 |
|  | Extreme phenotype differences | 79 | 679 | 332 |
| Height | Distribution | 532 | 4,246 | 2,246 |
|  | Phenotypic variability | 114 | 808 | 447 |
|  | Extreme phenotype differences | 409 | 3,232 | 1,692 |
| WC | Men | 148 | 1,335 | 715 |
|  | Women | 77 | 577 | 328 |
| WHR | Distribution | 139 | 1,246 | 702 |
|  | Extreme phenotype differences | 138 | 1,092 | 578 |
|  | Men | 65 | 515 | 257 |
|  | Women | 92 | 731 | 406 |
