# Supplementary material for: Evidence of Polygenic Adaptation in the Systems Genetics of Anthropometric Traits
Source: PLoS One. 2016 Aug 18;11(8):e0160654. doi: 10.1371/journal.pone.0160654 (PMC4990182; doi:10.1371/journal.pone.0160654)
Supplement: S3 Table — NI: not included in top-10 PPI modules. (DOCX) [file pone.0160654.s003.docx]

**S3 Table**: Genes and their correspondence p values present in gene network associated with BMI-related phenotypes. NI: not included in top-10 PPI modules.

| **Gene** | **Distribution** | **Phenotypic variability** | **Extreme phenotype differences** |
| --- | --- | --- | --- |
| *AATK* | 1.15E-03 | NI | NI |
| *ACSM5* | NI | NI | 7.50E-02 |
| *ACTR5* | NI | 4.22E-02 | NI |
| *ACTR8* | NI | 1.61E-02 | NI |
| *ADAMTS7* | 5.51E-02 | NI | NI |
| *ADAT3* | 2.00E-06 | NI | NI |
| *ADCY3* | <1.00E-06 | NI | 4.00E-06 |
| *ADPGK* | 1.18E-03 | 9.50E-01 | NI |
| *ADRB2* | 2.88E-01 | NI | NI |
| *AGBL2* | <1.00E-06 | NI | NI |
| *ANAPC7* | 1.71E-04 | NI | NI |
| *ANKRD26* | NI | 8.09E-03 | NI |
| *APC* | 1.86E-01 | NI | NI |
| *APEH* | NI | NI | 2.71E-02 |
| *APP* | 8.98E-01 | NI | 6.97E-01 |
| *ARHGDIB* | NI | 6.51E-04 | NI |
| *ARHGEF2* | 3.60E-05 | NI | NI |
| *ARNT* | NI | NI | 2.19E-03 |
| *ARNTL* | 2.88E-04 | NI | NI |
| *ARPC1A* | NI | 9.59E-01 | NI |
| *ARPC2* | 1.99E-01 | NI | NI |
| *ARPC3* | 1.78E-04 | NI | NI |
| *ARRB1* | NI | NI | 2.89E-01 |
| *ART4* | 4.84E-01 | NI | NI |
| *ATG12* | NI | 3.96E-02 | NI |
| *ATP2A2* | 4.50E-05 | NI | NI |
| *ATXN1* | 6.38E-02 | NI | NI |
| *BAG3* | 3.49E-01 | NI | NI |
| *BAIAP2* | 2.93E-03 | NI | NI |
| *BAK1* | NI | 5.41E-02 | NI |
| *BCAN* | 5.43E-01 | NI | NI |
| *BCL2L10* | NI | 1.19E-01 | NI |
| *BCL6* | NI | 9.20E-02 | NI |
| *BDNF* | <1.00E-06 | NI | 6.24E-04 |
| *BMPR2* | 9.57E-01 | NI | NI |
| *BNIP1* | NI | 5.45E-02 | NI |
| *BRCA1* | 5.78E-02 | NI | 5.47E-03 |
| *BRD9* | NI | NI | 2.43E-01 |
| *BTBD2* | NI | NI | 4.47E-04 |
| *BUB1B* | NI | 9.41E-03 | NI |
| *C1QBP* | 1.43E-04 | NI | NI |
| *C6orf106* | NI | NI | 5.86E-04 |
| *CA2* | NI | 1.26E-02 | NI |
| *CAMKK2* | NI | NI | 1.67E-03 |
| *CBL* | 1.58E-01 | NI | NI |
| *CBX1* | 1.07E-03 | NI | NI |
| *CBX4* | NI | 5.11E-01 | NI |
| *CCDC92* | NI | NI | 5.85E-01 |
| *CCND1* | NI | NI | 8.22E-03 |
| *CCNH* | 9.89E-03 | NI | NI |
| *CD58* | 5.35E-01 | NI | NI |
| *CD84* | NI | 4.95E-01 | NI |
| *CDC42* | NI | NI | 6.97E-02 |
| *CDC42EP3* | NI | NI | 2.17E-01 |
| *CEBPB* | 1.02E-01 | NI | NI |
| *CES3* | 8.12E-01 | NI | NI |
| *CHPF* | 1.23E-03 | NI | NI |
| *CKAP2L* | 8.68E-02 | NI | NI |
| *CLIP1* | 5.96E-03 | NI | NI |
| *CNDP1* | NI | 3.34E-02 | NI |
| *CNDP2* | NI | 3.38E-02 | NI |
| *COL4A3BP* | 2.00E-05 | NI | 4.79E-03 |
| *COL9A1* | 2.84E-01 | NI | NI |
| *COMP* | 2.07E-01 | NI | NI |
| *CRB2* | NI | 2.54E-04 | NI |
| *CREBBP* | 2.22E-01 | NI | 3.28E-02 |
| *CRNN* | 8.64E-01 | NI | NI |
| *CSDE1* | NI | 2.29E-02 | NI |
| *CSF2RB* | 2.08E-01 | NI | NI |
| *CSNK1E* | 1.16E-01 | NI | NI |
| *CSNK1G2* | 1.50E-05 | NI | 1.25E-04 |
| *CSNK2A1* | 9.38E-01 | NI | NI |
| *CSNK2B* | 6.47E-02 | NI | NI |
| *CUL3* | 1.40E-01 | 4.30E-01 | NI |
| *CWF19L2* | NI | NI | 7.44E-01 |
| *CYP20A1* | NI | NI | 5.02E-04 |
| *CYR61* | NI | NI | 1.85E-03 |
| *DAAM1* | NI | 2.71E-03 | NI |
| *DBNL* | NI | 2.84E-03 | NI |
| *DCUN1D1* | NI | 1.08E-02 | NI |
| *DDB2* | 2.90E-05 | NI | NI |
| *DDX17* | 9.43E-01 | NI | NI |
| *DDX6* | NI | 8.75E-03 | NI |
| *DGKG* | NI | NI | <1.00E-06 |
| *DHX57* | 9.52E-01 | NI | NI |
| *DLG1* | 1.83E-04 | NI | NI |
| *DNAJB4* | 1.01E-04 | NI | NI |
| *DNM1* | 7.49E-04 | NI | NI |
| *DNMT3B* | 3.93E-01 | NI | NI |
| *DYNC1I2* | 6.99E-03 | NI | NI |
| *ECT2* | 3.02E-03 | NI | NI |
| *EFEMP2* | NI | 5.23E-01 | NI |
| *EHMT2* | 1.18E-03 | NI | NI |
| *EIF3E* | 6.72E-02 | NI | NI |
| *ELAVL1* | 5.12E-01 | NI | 2.41E-01 |
| *EP300* | NI | NI | 3.69E-01 |
| *EPHB6* | NI | 3.48E-02 | NI |
| *ERP27* | NI | 6.32E-04 | NI |
| *ESR1* | 5.11E-01 | NI | NI |
| *ETV5* | <1.00E-06 | NI | <1.00E-06 |
| *EXOSC10* | 1.34E-03 | NI | NI |
| *FAM131C* | 7.51E-01 | NI | NI |
| *FAM49B* | 8.16E-01 | NI | NI |
| *FBXO17* | 6.81E-01 | NI | NI |
| *FBXO48* | 3.01E-01 | NI | NI |
| *FBXO6* | 3.02E-01 | NI | NI |
| *FCGR2C* | 6.94E-01 | NI | NI |
| *FLCN* | NI | NI | 9.59E-01 |
| *FN1* | 7.46E-02 | NI | NI |
| *FNBP4* | <1.00E-06 | NI | 6.50E-05 |
| *FTO* | <1.00E-06 | <1.00E-06 | <1.00E-06 |
| *FUBP1* | 1.32E-04 | NI | NI |
| *GAB2* | 4.50E-04 | NI | NI |
| *GNB2L1* | NI | 3.44E-02 | NI |
| *GNB4* | NI | NI | 9.81E-03 |
| *GNG2* | NI | NI | 9.81E-03 |
| *GOLGA2* | 1.13E-04 | NI | 4.06E-03 |
| *GPN3* | NI | 5.34E-01 | NI |
| *GRB2* | 5.76E-01 | NI | NI |
| *GRHL1* | NI | 5.38E-01 | NI |
| *GRHL3* | NI | 3.85E-01 | NI |
| *GRK5* | 3.37E-01 | NI | NI |
| *GRN* | 4.97E-02 | NI | NI |
| *GTF3A* | 4.00E-06 | NI | NI |
| *GUF1* | NI | 4.71E-03 | NI |
| *HAAO* | 5.93E-04 | NI | NI |
| *HDAC1* | 3.14E-01 | NI | NI |
| *HDAC3* | 3.93E-01 | NI | NI |
| *HERC2* | NI | NI | 1.04E-03 |
| *HERC4* | NI | 6.07E-01 | NI |
| *HIP1* | 5.00E-06 | NI | NI |
| *HIST1H4A* | NI | 7.06E-01 | NI |
| *HNRNPA0* | NI | 9.24E-03 | NI |
| *HOXA10* | NI | NI | 4.21E-03 |
| *HOXA11* | NI | NI | 4.28E-03 |
| *HOXA9* | NI | NI | 3.14E-03 |
| *HOXB3* | 2.07E-04 | NI | NI |
| *HSD17B4* | NI | 9.33E-03 | NI |
| *HSP90AA1* | 9.39E-02 | NI | NI |
| *HUS1B* | NI | NI | 3.66E-01 |
| *IDH2* | 3.48E-01 | NI | NI |
| *IL17RB* | NI | 1.69E-02 | NI |
| *IL21* | NI | NI | 4.46E-02 |
| *IL5* | 9.51E-01 | NI | NI |
| *IL5RA* | 1.14E-02 | NI | NI |
| *ING1* | NI | NI | 4.86E-01 |
| *IPO9* | 1.18E-04 | NI | NI |
| *IRF9* | NI | NI | 5.06E-01 |
| *JRK* | 1.40E-01 | NI | NI |
| *JUND* | 8.62E-01 | NI | NI |
| *KAT2A* | 8.97E-03 | NI | NI |
| *KAT2B* | NI | NI | 5.79E-03 |
| *KBTBD4* | <1.00E-06 | NI | NI |
| *KCNAB1* | NI | 5.86E-03 | NI |
| *KCTD15* | 2.00E-06 | NI | 5.90E-04 |
| *KCTD21* | NI | 2.38E-03 | NI |
| *KHDRBS1* | 6.99E-01 | NI | NI |
| *KIAA0556* | 2.03E-01 | NI | NI |
| *KIAA1598* | 5.01E-04 | NI | NI |
| *KIF13B* | NI | 6.15E-01 | NI |
| *KPNA1* | NI | NI | 2.42E-03 |
| *KRT14* | NI | 6.64E-03 | NI |
| *LDHA* | NI | 2.52E-01 | NI |
| *LEO1* | NI | 1.01E-02 | NI |
| *LMNA* | 1.36E-01 | NI | NI |
| *MADD* | <1.00E-06 | NI | 9.93E-04 |
| *MAGOH* | NI | 8.79E-03 | NI |
| *MANBA* | 4.08E-02 | NI | NI |
| *MAP1A* | 7.60E-05 | NI | NI |
| *MAP2* | 7.35E-01 | NI | NI |
| *MAP2K4* | NI | 1.03E-02 | NI |
| *MAP2K5* | 5.00E-06 | NI | 3.77E-03 |
| *MAP3K4* | 6.19E-01 | NI | NI |
| *MAP3K7* | NI | 8.17E-01 | NI |
| *MAP3K8* | NI | 1.68E-03 | NI |
| *MAPK1* | 3.24E-03 | NI | NI |
| *MAPK14* | NI | NI | 2.76E-02 |
| *MAPK6* | 2.33E-01 | NI | NI |
| *MAPK8* | 8.42E-02 | NI | NI |
| *MCC* | NI | NI | 9.77E-03 |
| *MCM3* | NI | 2.94E-03 | NI |
| *MCRS1* | NI | 4.46E-02 | NI |
| *METAP2* | NI | 9.03E-04 | NI |
| *MKI67* | 2.57E-01 | NI | NI |
| *MMP3* | 6.27E-01 | NI | NI |
| *MPST* | 7.20E-01 | NI | NI |
| *MRPL51* | 3.60E-01 | NI | NI |
| *MRPS15* | 8.49E-01 | NI | NI |
| *MTA3* | NI | NI | 2.05E-03 |
| *MTCH2* | <1.00E-06 | NI | 9.60E-05 |
| *MTIF3* | 2.00E-06 | NI | NI |
| *MYOZ1* | NI | NI | 4.16E-03 |
| *NAPA* | NI | 1.15E-01 | NI |
| *NCAM1* | 2.08E-03 | NI | NI |
| *NCAN* | 1.50E-02 | NI | NI |
| *NCL* | 6.23E-02 | NI | NI |
| *NDFIP2* | NI | 1.25E-03 | NI |
| *NDUFS3* | <1.00E-06 | NI | NI |
| *NEDD4L* | NI | 4.34E-02 | NI |
| *NFKB1* | 2.77E-01 | NI | NI |
| *NPM3* | 1.26E-03 | NI | NI |
| *NPPA* | NI | 9.64E-03 | NI |
| *NR1H3* | 2.20E-05 | NI | 7.55E-03 |
| *NR3C1* | NI | NI | 6.96E-02 |
| *NRF1* | 7.10E-01 | 1.83E-01 | NI |
| *NTRK2* | 1.40E-02 | NI | NI |
| *NUDT3* | 3.00E-06 | NI | NI |
| *NUP107* | 1.50E-01 | NI | NI |
| *NUP160* | <1.00E-06 | NI | NI |
| *NUP88* | 3.47E-04 | NI | NI |
| *OPA1* | 8.38E-01 | NI | NI |
| *PABPC4* | 5.72E-01 | NI | NI |
| *PACSIN1* | 8.10E-05 | NI | NI |
| *PAFAH1B1* | 3.96E-01 | NI | NI |
| *PARK2* | 1.01E-02 | NI | NI |
| *PARP2* | 7.75E-02 | NI | 1.69E-01 |
| *PCNA* | NI | 1.19E-02 | NI |
| *PELI2* | NI | 1.20E-02 | NI |
| *PHC2* | NI | 4.51E-02 | NI |
| *PIK3C3* | NI | 2.72E-04 | NI |
| *PIK3R2* | 1.76E-01 | NI | NI |
| *PKIA* | 4.71E-01 | NI | NI |
| *PLA2G4C* | NI | NI | 2.24E-01 |
| *PLCG1* | 8.23E-03 | NI | NI |
| *PLEKHA5* | NI | NI | 1.80E-01 |
| *PML* | 6.93E-01 | NI | NI |
| *POLM* | NI | 2.73E-03 | NI |
| *POM121C* | <1.00E-06 | NI | NI |
| *POT1* | 1.14E-01 | NI | NI |
| *POU5F1* | 4.89E-03 | NI | NI |
| *PPP1CC* | 6.65E-03 | NI | NI |
| *PPP3CC* | 9.01E-01 | NI | NI |
| *PRDX3* | 6.50E-01 | NI | NI |
| *PREPL* | NI | NI | 1.50E-02 |
| *PRKAA2* | NI | NI | 2.59E-01 |
| *PRMT5* | NI | 1.58E-01 | NI |
| *PSMC3* | <1.00E-06 | NI | NI |
| *PTBP2* | NI | NI | 1.17E-03 |
| *PTPMT1* | <1.00E-06 | NI | NI |
| *PTPN11* | 1.50E-04 | NI | NI |
| *PTPRN* | 2.34E-02 | NI | NI |
| *RABEP1* | 1.76E-04 | NI | 6.76E-04 |
| *RAD9A* | NI | NI | 1.42E-01 |
| *RAE1* | 4.61E-01 | NI | NI |
| *RAPSN* | <1.00E-06 | NI | NI |
| *RARB* | 3.40E-01 | 9.14E-01 | NI |
| *RASL11B* | NI | 1.75E-02 | NI |
| *RB1CC1* | NI | 1.14E-02 | NI |
| *RBM39* | 7.84E-01 | NI | NI |
| *RFWD2* | 1.65E-02 | NI | 5.10E-03 |
| *RNF19A* | NI | 6.35E-03 | NI |
| *RNMTL1* | NI | NI | 2.03E-02 |
| *RPL27* | NI | NI | 6.72E-03 |
| *RPL6* | 1.30E-04 | NI | NI |
| *RPS10* | <1.00E-06 | NI | 9.15E-04 |
| *RPS18* | 2.31E-02 | NI | NI |
| *RPS2* | 9.10E-03 | NI | NI |
| *RPS25* | 1.29E-01 | NI | NI |
| *RPS27L* | NI | 1.96E-02 | NI |
| *RPS3A* | NI | NI | 2.30E-02 |
| *RXRB* | NI | 1.02E-01 | NI |
| *S100A10* | NI | NI | 6.01E-02 |
| *SAMD4B* | 6.14E-01 | NI | NI |
| *SCG5* | NI | 1.38E-02 | NI |
| *SEC13* | NI | NI | 3.69E-01 |
| *SEC16B* | NI | NI | <1.00E-06 |
| *SERTAD2* | NI | NI | 6.15E-03 |
| *SETDB1* | NI | NI | 6.11E-03 |
| *SFXN2* | NI | 3.01E-03 | NI |
| *SHMT1* | NI | NI | 1.13E-01 |
| *SIAE* | 2.11E-01 | NI | NI |
| *SIN3A* | 1.91E-01 | NI | NI |
| *SIRT1* | 2.03E-04 | NI | NI |
| *SLC27A4* | 8.80E-05 | NI | NI |
| *SLC43A1* | NI | 1.05E-01 | NI |
| *SLC9A3R1* | NI | 1.09E-01 | 1.44E-01 |
| *SMAD3* | 1.08E-02 | NI | NI |
| *SMG1* | NI | 1.16E-02 | NI |
| *SPCS2* | 2.15E-03 | NI | NI |
| *SPI1* | <1.00E-06 | NI | 1.06E-03 |
| *SPOCK1* | 6.23E-02 | NI | NI |
| *SPOCK3* | 4.35E-02 | NI | NI |
| *SPTAN1* | NI | NI | 1.26E-02 |
| *SREBF2* | 4.45E-02 | NI | NI |
| *STK17A* | NI | 3.10E-01 | NI |
| *STK25* | 1.90E-01 | NI | NI |
| *STX18* | NI | 5.68E-02 | NI |
| *SUFU* | NI | 2.93E-03 | NI |
| *SUMO2* | 9.47E-03 | NI | 7.60E-01 |
| *TAL1* | 1.50E-04 | NI | 4.66E-03 |
| *TARDBP* | 3.16E-03 | NI | NI |
| *TCF7L2* | 2.40E-04 | NI | NI |
| *TELO2* | NI | 1.10E-02 | NI |
| *TFAP2B* | 2.00E-06 | NI | NI |
| *THG1L* | NI | 4.19E-02 | NI |
| *TMED5* | NI | 2.97E-01 | NI |
| *TMSB10* | 5.54E-01 | NI | NI |
| *TNF* | 9.66E-03 | NI | NI |
| *TNFRSF14* | 1.21E-04 | NI | NI |
| *TNR* | 2.26E-01 | NI | NI |
| *TNRC6A* | NI | 7.60E-04 | NI |
| *TNRC6B* | 1.29E-04 | NI | NI |
| *TOP1* | 7.04E-03 | NI | NI |
| *TOP3B* | 1.59E-03 | NI | NI |
| *TP53* | 1.96E-01 | NI | NI |
| *TP53BP1* | 7.80E-05 | 4.91E-01 | NI |
| *TP63* | 5.65E-02 | NI | NI |
| *TRIM33* | NI | 2.31E-02 | NI |
| *TRIM8* | NI | 3.99E-03 | NI |
| *TRPC3* | NI | 3.88E-02 | NI |
| *TRPV1* | 5.80E-01 | NI | NI |
| *TUBA4A* | 9.13E-03 | NI | NI |
| *TUBGCP4* | 2.76E-03 | NI | NI |
| *UBAP2* | 2.23E-04 | NI | NI |
| *UBC* | 5.15E-01 | 5.45E-01 | 8.28E-01 |
| *UBD* | 6.07E-02 | 2.35E-01 | NI |
| *UBE2B* | NI | 1.57E-02 | NI |
| *UBE2Q2* | 3.01E-01 | NI | NI |
| *UBE2R2* | 2.37E-04 | NI | NI |
| *UBQLN4* | 1.01E-04 | 1.11E-01 | NI |
| *UGP2* | NI | 6.21E-03 | NI |
| *UPF1* | 2.01E-01 | NI | NI |
| *VAMP2* | NI | 1.06E-01 | NI |
| *VAV1* | 9.31E-01 | NI | NI |
| *VIL1* | 6.65E-04 | NI | NI |
| *VPS29* | 1.10E-04 | NI | NI |
| *VPS4B* | NI | 1.86E-03 | NI |
| *WBP2* | NI | 1.92E-02 | NI |
| *WDR59* | 9.91E-01 | NI | NI |
| *WWOX* | 3.56E-01 | NI | NI |
| *XPO1* | 3.52E-01 | NI | NI |
| *YEATS2* | NI | 6.32E-03 | NI |
| *YKT6* | 8.91E-02 | NI | NI |
| *YWHAG* | 1.56E-02 | NI | NI |
| *YWHAZ* | 2.88E-02 | NI | 6.62E-03 |
| *ZBTB10* | 1.85E-04 | NI | NI |
| *ZC3H4* | NI | NI | 5.96E-01 |
