# Supplementary material for: Evidence of Polygenic Adaptation in the Systems Genetics of Anthropometric Traits
Source: PLoS One. 2016 Aug 18;11(8):e0160654. doi: 10.1371/journal.pone.0160654 (PMC4990182; doi:10.1371/journal.pone.0160654)
Supplement: S4 Table — (DOCX) [file pone.0160654.s004.docx]

**S4 Table**: Significant long-distance genotypic LDs observed in BMI-associated gene networks.

| **rsId1** | **chr1** | **loc1** | **rsId2** | **chr2** | **loc2** | **p.value** | **q.value** | **SNP1_p** | **SNP1_iHS** | **SNP2_p** | **SNP2_iHS** | **Gene1** | **Gene1_p** | **Gene2** | **Gene2_p** | **BMI-related phenotype** |
| --- | --- | --- | --- | --- | --- | --- | --- | --- | --- | --- | --- | --- | --- | --- | --- | --- |
| rs3917925 | 1 | 36718146 | rs7853999 | 9 | 130186230 | 2.00E-06 | 4.09E-02 | 6.49E-01 | 1.83 | 7.54E-02 | 1.70 | *MRPS15* | 8.49E-01 | *SLC27A4* | 8.80E-05 | Distribution |
| rs2494248 | 1 | 47417489 | rs17389644 | 4 | 123717147 | < 1.00E-06 | < 4.09E-02 | 1.10E-01 | 1.85 | 3.40E-01 | -1.67 | *PDZK1IP1* | 2.54E-03 | *IL21* | 4.46E-02 | Extreme phenotype differences |
| rs10858047 | 1 | 114875399 | rs6818178 | 4 | 53419501 | < 1.00E-06 | < 4.09E-02 | 5.60E-03 | -1.50 | 1.80E-02 | 1.59 | *BCAS2* | 1.80E-02 | *RASL11B* | 1.75E-02 | Phenotypic variability |
| rs1059823 | 2 | 218968088 | rs11641340 | 16 | 77109437 | < 1.00E-06 | < 4.09E-02 | 2.39E-02 | -1.51 | 3.33E-01 | 2.77 | *C2orf62* | 1.65E-02 | *WWOX* | 3.56E-01 | Distribution |
| rs6768285 | 3 | 25518172 | rs578210 | 8 | 81528891 | < 1.00E-06 | < 4.09E-02 | 7.98E-01 | 1.67 | 2.75E-02 | -1.67 | *RARB* | 3.40E-01 | *ZBTB10* | 1.85E-04 | Distribution |
| rs13317328 | 3 | 53845880 | rs13190347 | 5 | 157134118 | < 1.00E-06 | < 4.09E-02 | 4.60E-01 | 2.29 | 8.40E-01 | -1.70 | *SELK* | 1.35E-02 | *THG1L* | 4.19E-02 | Phenotypic variability |
| rs7672268 | 4 | 103771906 | rs9930506 | 16 | 52387966 | < 1.00E-06 | < 4.09E-02 | 9.69E-02 | -1.78 | 1.41E-53 | 1.65 | *MANBA* | 4.08E-02 | *FTO* | < 1.00E-06 | Distribution |
| rs186543 | 5 | 86790591 | rs16948137 | 16 | 77180189 | 2.00E-06 | 4.09E-02 | 3.84E-03 | -1.52 | 4.69E-02 | 3.17 | *CCNH* | 9.89E-03 | *WWOX* | 3.56E-01 | Distribution |
| rs454886 | 5 | 112174016 | rs10945756 | 6 | 161910329 | < 1.00E-06 | < 4.09E-02 | 1.51E-01 | -1.67 | 5.34E-01 | -2.13 | *APC* | 1.86E-01 | *PARK2* | 1.01E-02 | Distribution |
| rs1997716 | 6 | 16708658 | rs10971796 | 9 | 33915676 | < 1.00E-06 | < 4.09E-02 | 7.38E-02 | 1.69 | 6.71E-01 | -1.51 | *ATXN1* | 6.38E-02 | *UBAP2* | 2.23E-04 | Distribution |
| rs1233386 | 6 | 29666169 | rs17618704 | 17 | 43576009 | < 1.00E-06 | < 4.09E-02 | 6.44E-03 | -2.00 | 1.93E-02 | -3.10 | *OR2H2* | 2.48E-02 | *SNX11* | 3.94E-04 | Distribution |
| rs805284 | 6 | 31790008 | rs11648121 | 16 | 76739487 | < 1.00E-06 | < 4.09E-02 | 8.88E-01 | -2.61 | 3.40E-01 | 1.73 | *BAT5* | 4.37E-02 | *WWOX* | 3.56E-01 | Distribution |
| rs805284 | 6 | 31790008 | rs7501067 | 16 | 76767164 | 2.00E-06 | 4.09E-02 | 8.88E-01 | -2.61 | 2.23E-01 | -1.54 | *BAT5* | 4.37E-02 | *WWOX* | 3.56E-01 | Distribution |
| rs3780632 | 9 | 86542608 | rs2502731 | 9 | 130016378 | 2.00E-06 | 4.09E-02 | 1.06E-01 | -1.85 | 9.36E-02 | 2.33 | *NTRK2* | 1.40E-02 | *GOLGA2* | 1.13E-04 | Distribution |
| rs10116453 | 9 | 86553021 | rs3003608 | 9 | 130024251 | 2.00E-06 | 4.09E-02 | 5.13E-02 | 1.87 | 3.48E-02 | -2.07 | *NTRK2* | 1.40E-02 | *GOLGA2* | 1.13E-04 | Distribution |
| rs4734 | 9 | 130058460 | rs2829946 | 21 | 26138228 | < 1.00E-06 | < 4.09E-02 | 3.53E-03 | 1.80 | 8.32E-01 | -1.65 | *C9orf119* | 4.40E-05 | *APP* | 8.98E-01 | Distribution |
| rs4734 | 9 | 130058460 | rs2829946 | 21 | 26138228 | 2.00E-06 | 4.91E-02 | 2.20E-02 | 1.80 | 6.40E-01 | -1.65 | *C9orf119* | 2.52E-03 | *APP* | 6.97E-01 | Extreme phenotype differences |
| rs2079867 | 12 | 6492372 | rs12603094 | 17 | 5288680 | 2.00E-06 | 4.09E-02 | 6.63E-01 | 2.31 | 3.73E-01 | 2.38 | *IFFO* | 3.24E-01 | *DHX33* | 9.20E-05 | Distribution |
| rs7975712 | 12 | 48191980 | rs12439200 | 15 | 70873784 | < 1.00E-06 | < 4.09E-02 | 3.80E-02 | -2.20 | 7.50E-01 | 2.05 | *MCRS1* | 4.46E-02 | *ADPGK* | 9.50E-01 | Phenotypic variability |
| rs7157967 | 14 | 101655966 | rs11629568 | 15 | 65269422 | < 1.00E-06 | < 4.09E-02 | 1.33E-01 | 2.43 | 1.22E-01 | -1.83 | *HSP90AA1* | 9.39E-02 | *SMAD3* | 1.08E-02 | Distribution |
| rs1190583 | 14 | 101679429 | rs11629568 | 15 | 65269422 | < 1.00E-06 | < 4.09E-02 | 2.04E-01 | 2.43 | 1.22E-01 | -1.83 | *HSP90AA1* | 9.39E-02 | *SMAD3* | 1.08E-02 | Distribution |
| rs1992215 | 15 | 65205991 | rs12917864 | 16 | 76959503 | < 1.00E-06 | < 4.09E-02 | 4.82E-02 | 1.80 | 2.97E-01 | -1.88 | *SMAD3* | 1.08E-02 | *WWOX* | 3.56E-01 | Distribution |
| rs1992215 | 15 | 65205991 | rs13337989 | 16 | 76964954 | < 1.00E-06 | < 4.09E-02 | 4.82E-02 | 1.80 | 1.06E-01 | -1.73 | *SMAD3* | 1.08E-02 | *WWOX* | 3.56E-01 | Distribution |
| rs1878699 | 15 | 65687937 | rs13056733 | 22 | 38976018 | < 1.00E-06 | < 4.09E-02 | 2.17E-04 | -1.64 | 5.95E-02 | -2.49 | *MAP2K5* | 5.00E-06 | *TNRC6B* | 1.29E-04 | Distribution |
| rs3784716 | 15 | 65871504 | rs13056733 | 22 | 38976018 | < 1.00E-06 | < 4.09E-02 | 5.17E-04 | -1.90 | 5.95E-02 | -2.49 | *MAP2K5* | 5.00E-06 | *TNRC6B* | 1.29E-04 | Distribution |
