# Supplementary material for: Evidence of Polygenic Adaptation in the Systems Genetics of Anthropometric Traits
Source: PLoS One. 2016 Aug 18;11(8):e0160654. doi: 10.1371/journal.pone.0160654 (PMC4990182; doi:10.1371/journal.pone.0160654)
Supplement: S5 Table — NI: not included in top-10 PPI modules. (DOCX) [file pone.0160654.s005.docx]

**S5 Table**: Genes and their correspondence p values present in gene network associated with height-related phenotypes. NI: not included in top-10 PPI modules.

| **Gene** | **Distribution** | **Phenotypic.variability** | **Extreme.phenotype.differences** |
| --- | --- | --- | --- |
| *AANAT* | NI | 3.90E-02 | NI |
| *ABL1* | 5.37E-01 | NI | NI |
| *ACAN* | 9.00E-06 | NI | NI |
| *ACIN1* | 1.04E-02 | NI | NI |
| *ACP6* | NI | NI | 3.64E-02 |
| *ACSS2* | < 1.00E-06 | NI | NI |
| *ACTN3* | 1.29E-01 | NI | NI |
| *ADAMTS1* | 7.36E-02 | NI | NI |
| *ADCY3* | < 1.00E-06 | NI | < 1.00E-06 |
| *ADCY4* | < 1.00E-06 | NI | NI |
| *ADRA2B* | 7.22E-01 | NI | NI |
| *AIF1* | < 1.00E-06 | NI | NI |
| *AKR1C1* | 9.95E-04 | NI | NI |
| *ALB* | 2.48E-01 | NI | NI |
| *ALPP* | < 1.00E-06 | NI | NI |
| *ANKIB1* | NI | NI | < 1.00E-06 |
| *ANP32E* | < 1.00E-06 | NI | NI |
| *AP3D1* | 2.00E-06 | NI | NI |
| *APC* | 2.45E-02 | NI | NI |
| *APOA1* | 1.44E-02 | NI | NI |
| *APOA2* | 9.26E-01 | NI | NI |
| *APOE* | 1.93E-02 | NI | NI |
| *APOH* | 1.58E-02 | NI | NI |
| *APP* | 7.90E-02 | NI | 7.49E-02 |
| *APRT* | 5.80E-05 | NI | < 1.00E-06 |
| *ARCN1* | 2.49E-04 | NI | NI |
| *ARFGEF2* | < 1.00E-06 | NI | NI |
| *ARHGDIB* | 4.56E-01 | NI | NI |
| *ARL2* | 1.08E-03 | NI | NI |
| *ARL2BP* | 3.59E-02 | NI | NI |
| *ARNT* | 4.30E-01 | NI | NI |
| *ATF7IP2* | 5.20E-01 | NI | NI |
| *ATP13A2* | < 1.00E-06 | 5.67E-01 | NI |
| *ATP4A* | NI | 1.69E-01 | NI |
| *ATP6V1G2* | < 1.00E-06 | NI | NI |
| *ATP6V1H* | 9.29E-01 | NI | NI |
| *ATP7B* | 9.55E-01 | NI | NI |
| *BAG2* | NI | 1.16E-02 | NI |
| *BAG3* | 1.27E-01 | 1.45E-01 | NI |
| *BAG5* | 4.41E-03 | NI | NI |
| *BANP* | 1.38E-02 | NI | NI |
| *BATF2* | 1.13E-03 | NI | NI |
| *BCAS2* | NI | 1.02E-02 | NI |
| *BCAS3* | < 1.00E-06 | NI | < 1.00E-06 |
| *BCL2L1* | 1.04E-02 | NI | NI |
| *BCS1L* | 3.00E-06 | NI | NI |
| *BEGAIN* | 4.72E-01 | NI | NI |
| *BLK* | 4.63E-03 | NI | NI |
| *BLVRB* | 2.81E-01 | NI | NI |
| *BMI1* | 4.54E-02 | NI | NI |
| *BMPR1B* | 4.39E-01 | NI | NI |
| *BTN3A1* | 3.89E-01 | NI | NI |
| *BTN3A3* | < 1.00E-06 | NI | NI |
| *C11orf45* | 1.05E-01 | NI | NI |
| *C17orf82* | < 1.00E-06 | NI | < 1.00E-06 |
| *C18orf32* | < 1.00E-06 | NI | NI |
| *C2* | < 1.00E-06 | NI | NI |
| *C2orf49* | NI | 1.92E-02 | NI |
| *C4orf27* | 7.43E-02 | NI | NI |
| *C6orf15* | < 1.00E-06 | NI | NI |
| *C6orf47* | < 1.00E-06 | NI | NI |
| *C7orf25* | 3.46E-01 | NI | NI |
| *C9orf78* | NI | 2.15E-02 | NI |
| *CA11* | 5.79E-01 | NI | NI |
| *CABLES1* | < 1.00E-06 | NI | NI |
| *CALM1* | 5.18E-01 | NI | NI |
| *CAMK2B* | 3.52E-01 | NI | NI |
| *CAMK2G* | NI | NI | 2.98E-02 |
| *CAMKK2* | 6.46E-01 | NI | NI |
| *CASKIN1* | 1.20E-05 | NI | NI |
| *CAV1* | NI | 3.42E-03 | 3.31E-01 |
| *CAV2* | NI | 4.38E-03 | NI |
| *CCDC116* | 9.81E-01 | NI | NI |
| *CCDC47* | < 1.00E-06 | NI | NI |
| *CCDC91* | < 1.00E-06 | NI | NI |
| *CCHCR1* | 4.00E-06 | NI | NI |
| *CCK* | 2.97E-01 | NI | NI |
| *CCNA1* | NI | 2.29E-02 | NI |
| *CCNO* | NI | 6.12E-03 | NI |
| *CCT5* | 4.82E-01 | NI | NI |
| *CD47* | NI | 1.83E-01 | NI |
| *CDCA3* | 1.28E-01 | NI | NI |
| *CDK5RAP3* | 8.00E-06 | NI | NI |
| *CDK6* | < 1.00E-06 | NI | < 1.00E-06 |
| *CDT1* | NI | NI | < 1.00E-06 |
| *CEBPE* | NI | 1.56E-03 | NI |
| *CEBPG* | NI | 1.43E-02 | NI |
| *CENPJ* | NI | 5.61E-03 | NI |
| *CEP250* | < 1.00E-06 | NI | < 1.00E-06 |
| *CEP63* | 2.30E-03 | NI | NI |
| *CFB* | < 1.00E-06 | NI | NI |
| *CGB* | NI | NI | 5.09E-01 |
| *CHCHD6* | 5.49E-01 | NI | NI |
| *CHD3* | 1.10E-01 | NI | NI |
| *CLEC4G* | NI | 1.23E-02 | NI |
| *CLU* | 1.07E-01 | NI | NI |
| *CNKSR1* | 7.30E-05 | NI | NI |
| *COL11A1* | 2.00E-06 | NI | NI |
| *COL3A1* | 8.82E-02 | NI | NI |
| *COMMD7* | 1.46E-03 | NI | NI |
| *COMMD8* | 4.58E-01 | NI | NI |
| *COPZ2* | 4.40E-05 | NI | NI |
| *CORT* | NI | 7.75E-01 | NI |
| *CPNE1* | < 1.00E-06 | NI | < 1.00E-06 |
| *CPSF4* | 5.46E-02 | NI | NI |
| *CPVL* | NI | 3.97E-01 | NI |
| *CRCT1* | 6.60E-01 | NI | NI |
| *CREB3* | NI | 1.89E-01 | NI |
| *CREBBP* | 6.38E-01 | NI | NI |
| *CRK* | 7.24E-01 | NI | NI |
| *CRMP1* | 1.31E-01 | NI | NI |
| *CROCC* | NI | NI | < 1.00E-06 |
| *CSE1L* | 5.30E-05 | NI | NI |
| *CSF2* | NI | NI | < 1.00E-06 |
| *CSN3* | 6.37E-01 | NI | NI |
| *CSNK2B* | < 1.00E-06 | NI | NI |
| *CTDSP1* | < 1.00E-06 | NI | NI |
| *CTNNB1* | 4.20E-02 | 2.37E-02 | NI |
| *CTRL* | 2.83E-02 | NI | NI |
| *CTSB* | 8.67E-01 | NI | NI |
| *CUL1* | NI | 3.86E-01 | NI |
| *CUL3* | 2.61E-02 | NI | 1.72E-02 |
| *CUL4A* | 1.39E-01 | NI | NI |
| *CUL5* | 7.92E-01 | NI | NI |
| *CUX1* | NI | 7.24E-01 | NI |
| *CXCR4* | NI | 8.95E-01 | NI |
| *DCP1A* | NI | 6.30E-03 | NI |
| *DDB2* | 1.07E-02 | NI | NI |
| *DDHD2* | NI | NI | 3.85E-01 |
| *DDR1* | 1.30E-04 | NI | NI |
| *DDX1* | NI | 2.65E-02 | NI |
| *DDX42* | < 1.00E-06 | NI | < 1.00E-06 |
| *DDX58* | 7.77E-01 | NI | NI |
| *DEF6* | < 1.00E-06 | NI | NI |
| *DIS3L2* | < 1.00E-06 | NI | NI |
| *DNAJC5* | NI | 4.37E-03 | NI |
| *DNTT* | 9.47E-01 | NI | NI |
| *DOHH* | < 1.00E-06 | NI | NI |
| *DOLK* | NI | 5.40E-01 | NI |
| *DOT1L* | < 1.00E-06 | NI | NI |
| *DTL* | 1.10E-05 | NI | NI |
| *DYM* | < 1.00E-06 | NI | < 1.00E-06 |
| *DYNLRB1* | < 1.00E-06 | NI | NI |
| *DZIP3* | 7.58E-02 | NI | NI |
| *E2F1* | < 1.00E-06 | NI | NI |
| *EBF4* | 8.81E-01 | NI | NI |
| *EDEM2* | < 1.00E-06 | NI | < 1.00E-06 |
| *EED* | 1.02E-01 | NI | NI |
| *EFCAB6* | 6.49E-02 | NI | NI |
| *EHMT2* | < 1.00E-06 | NI | < 1.00E-06 |
| *EIF3K* | 1.83E-01 | NI | NI |
| *EIF6* | < 1.00E-06 | 2.50E-03 | < 1.00E-06 |
| *ELAVL1* | 6.18E-01 | NI | NI |
| *ELP2* | 2.70E-02 | NI | NI |
| *EMCN* | 4.35E-01 | NI | NI |
| *ENO1* | 8.00E-01 | NI | NI |
| *EP300* | 2.24E-01 | NI | NI |
| *ERGIC3* | < 1.00E-06 | NI | < 1.00E-06 |
| *ERO1L* | 4.73E-02 | NI | NI |
| *ESR1* | 1.48E-04 | NI | 2.91E-04 |
| *ETHE1* | 4.06E-03 | NI | NI |
| *ETV5* | NI | 2.47E-04 | NI |
| *EWSR1* | 5.18E-02 | NI | 1.47E-01 |
| *FABP3* | NI | 5.90E-02 | NI |
| *FAM133B* | < 1.00E-06 | NI | < 1.00E-06 |
| *FAM63A* | NI | 6.68E-03 | NI |
| *FAM83C* | < 1.00E-06 | NI | < 1.00E-06 |
| *FAM8A1* | NI | 2.90E-05 | NI |
| *FANCC* | 2.61E-02 | NI | NI |
| *FANCE* | < 1.00E-06 | NI | NI |
| *FARP2* | < 1.00E-06 | NI | NI |
| *FBP1* | 1.22E-04 | NI | NI |
| *FBXL22* | 2.77E-01 | NI | NI |
| *FBXO6* | 3.25E-01 | NI | 1.39E-01 |
| *FBXW10* | 1.67E-01 | NI | NI |
| *FGD3* | 1.47E-01 | NI | NI |
| *FGFR4* | < 1.00E-06 | NI | NI |
| *FHOD1* | 2.08E-02 | NI | NI |
| *FKBPL* | < 1.00E-06 | NI | NI |
| *FN1* | 4.85E-02 | NI | 1.55E-02 |
| *FNDC3B* | < 1.00E-06 | NI | < 1.00E-06 |
| *FOXO3* | NI | NI | 5.45E-02 |
| *FRK* | 3.71E-04 | NI | NI |
| *FRS2* | 2.70E-05 | NI | NI |
| *FTSJ3* | < 1.00E-06 | NI | NI |
| *FUT8* | 2.69E-01 | NI | NI |
| *FXR2* | 5.43E-02 | NI | NI |
| *GAB1* | 4.37E-04 | NI | NI |
| *GABBR1* | 6.07E-03 | NI | NI |
| *GAMT* | 4.01E-01 | NI | NI |
| *GAPDH* | 8.46E-03 | NI | NI |
| *GATAD1* | < 1.00E-06 | NI | < 1.00E-06 |
| *GDF5* | < 1.00E-06 | NI | NI |
| *GEMIN4* | 8.21E-03 | NI | NI |
| *GGT7* | < 1.00E-06 | NI | NI |
| *GH1* | < 1.00E-06 | NI | < 1.00E-06 |
| *GLS* | 4.26E-04 | NI | NI |
| *GMDS* | 1.59E-02 | 6.80E-03 | NI |
| *GNA12* | < 1.00E-06 | NI | < 1.00E-06 |
| *GNA13* | NI | 1.70E-02 | NI |
| *GNAI2* | 1.04E-01 | NI | NI |
| *GNAS* | 6.14E-02 | NI | NI |
| *GNB2* | NI | 3.63E-01 | NI |
| *GNB5* | NI | 1.96E-02 | NI |
| *GNG7* | NI | NI | 7.85E-01 |
| *GNGT1* | 5.90E-01 | NI | NI |
| *GNRH1* | 3.70E-02 | NI | NI |
| *GOLGA2* | 4.71E-03 | NI | NI |
| *GP2* | NI | 2.59E-01 | NI |
| *GPR126* | < 1.00E-06 | NI | < 1.00E-06 |
| *GPSM1* | 1.75E-03 | NI | NI |
| *GPSM2* | 6.71E-01 | NI | NI |
| *GRAMD1C* | 4.44E-01 | NI | NI |
| *GRAMD4* | NI | 2.47E-04 | NI |
| *GRB2* | 2.15E-03 | NI | 8.29E-02 |
| *GRK5* | 7.76E-04 | NI | NI |
| *GSS* | < 1.00E-06 | NI | NI |
| *GTF2B* | 1.05E-04 | NI | NI |
| *GTF2F2* | NI | 3.45E-02 | NI |
| *GZMA* | NI | 1.70E-02 | NI |
| *H2AFV* | NI | 8.74E-03 | NI |
| *HAPLN1* | 8.14E-01 | NI | NI |
| *HAX1* | NI | 7.52E-01 | NI |
| *HCP5* | < 1.00E-06 | NI | NI |
| *HDAC1* | 9.24E-01 | NI | 3.52E-02 |
| *HDAC11* | 2.30E-01 | 1.57E-01 | 6.33E-02 |
| *HDAC5* | NI | NI | 4.92E-02 |
| *HDGF* | NI | NI | 8.29E-01 |
| *HDLBP* | < 1.00E-06 | NI | NI |
| *HEATR1* | NI | 1.75E-03 | NI |
| *HECW2* | 6.43E-01 | NI | NI |
| *HHIP* | < 1.00E-06 | NI | < 1.00E-06 |
| *HIST1H1A* | 5.00E-06 | NI | 3.40E-05 |
| *HIST1H1C* | < 1.00E-06 | NI | NI |
| *HIST1H1E* | NI | NI | 2.50E-05 |
| *HIST1H3A* | 8.00E-06 | NI | 2.40E-05 |
| *HIST1H4A* | 3.00E-06 | NI | 2.30E-05 |
| *HLA-B* | < 1.00E-06 | NI | 4.60E-05 |
| *HLA-C* | < 1.00E-06 | NI | < 1.00E-06 |
| *HMG20B* | 2.31E-01 | NI | NI |
| *HMGA1* | < 1.00E-06 | NI | < 1.00E-06 |
| *HNRNPK* | 4.00E-06 | NI | 7.90E-05 |
| *HOMER2* | 5.44E-01 | NI | NI |
| *HOXB13* | 2.44E-02 | NI | NI |
| *HOXB4* | 1.00E-04 | NI | NI |
| *HOXD11* | 8.70E-01 | NI | NI |
| *HOXD4* | 5.63E-01 | NI | NI |
| *HP1BP3* | NI | 8.85E-03 | NI |
| *HPGD* | 9.40E-01 | NI | NI |
| *HRK* | 3.55E-03 | NI | NI |
| *HSP90AA1* | 5.17E-01 | NI | NI |
| *HSP90AB1* | 8.70E-01 | NI | NI |
| *HSPA1A* | < 1.00E-06 | NI | 8.00E-06 |
| *HSPA1L* | 7.00E-06 | NI | NI |
| *HSPA4* | NI | 4.23E-02 | NI |
| *HTATIP2* | 9.92E-01 | NI | NI |
| *IARS* | 1.60E-05 | NI | NI |
| *IDI1* | 6.08E-01 | NI | NI |
| *IGF1* | 1.40E-01 | NI | NI |
| *IGF2BP1* | NI | 7.35E-01 | NI |
| *IGFBP1* | 5.88E-01 | NI | NI |
| *IGFBP5* | 6.71E-01 | NI | NI |
| *IL17A* | 4.89E-01 | NI | NI |
| *IL1B* | NI | NI | 8.05E-02 |
| *IL3* | NI | NI | < 1.00E-06 |
| *ILK* | 1.05E-01 | NI | NI |
| *ING1* | 7.64E-02 | NI | NI |
| *IQGAP1* | 7.92E-02 | NI | NI |
| *IQGAP3* | 2.61E-02 | NI | NI |
| *IRF1* | 1.00E-06 | NI | < 1.00E-06 |
| *IRF9* | 1.20E-02 | NI | NI |
| *ITCH* | 8.40E-05 | NI | NI |
| *ITGA8* | NI | 6.63E-01 | NI |
| *ITPR3* | 1.00E-06 | NI | NI |
| *JARID2* | 1.00E-01 | NI | NI |
| *JOSD1* | 2.62E-02 | NI | NI |
| *JRK* | 4.92E-02 | NI | NI |
| *KAT2A* | NI | NI | 7.56E-02 |
| *KCNK18* | 9.26E-01 | NI | NI |
| *KCNQ5* | 7.99E-01 | NI | NI |
| *KIAA0391* | NI | 4.10E-05 | NI |
| *KIF1B* | NI | NI | 8.13E-02 |
| *KLC1* | 3.46E-04 | NI | NI |
| *KLC2* | 3.10E-05 | NI | NI |
| *KRT15* | 4.67E-03 | NI | NI |
| *KRT31* | 1.24E-01 | NI | NI |
| *KRT6B* | 6.71E-01 | NI | NI |
| *KRTAP4-12* | 3.94E-02 | NI | NI |
| *LCOR* | 1.40E-01 | NI | NI |
| *LGALS8* | NI | 1.11E-03 | NI |
| *LIMD2* | < 1.00E-06 | NI | < 1.00E-06 |
| *LIMS1* | NI | NI | 1.28E-01 |
| *LMCD1* | 3.64E-01 | NI | NI |
| *LMF1* | 7.93E-04 | NI | NI |
| *LMNA* | 4.94E-01 | NI | NI |
| *LRRC20* | 6.19E-01 | NI | NI |
| *LSM2* | 3.30E-05 | NI | NI |
| *LSM8* | NI | 1.26E-02 | NI |
| *LTA* | < 1.00E-06 | NI | 1.48E-04 |
| *LTA4H* | 6.50E-01 | NI | NI |
| *LTB* | < 1.00E-06 | NI | 8.50E-05 |
| *LYN* | 7.50E-05 | NI | NI |
| *MAGI2* | 9.47E-01 | NI | NI |
| *MAGOH* | NI | NI | 3.60E-01 |
| *MAP1LC3A* | < 1.00E-06 | NI | NI |
| *MAP3K3* | < 1.00E-06 | 2.49E-01 | < 1.00E-06 |
| *MAP3K5* | 9.12E-02 | NI | NI |
| *MAPK8IP3* | 1.94E-01 | NI | NI |
| *MAPRE1* | 2.15E-03 | NI | NI |
| *MAPT* | 2.08E-03 | NI | NI |
| *MARCH3* | 6.57E-01 | NI | NI |
| *MARS* | 5.04E-02 | NI | NI |
| *MASP1* | 2.79E-01 | NI | NI |
| *MASP2* | 6.25E-04 | NI | NI |
| *MAX* | 1.55E-03 | NI | NI |
| *MCAT* | 9.70E-01 | NI | NI |
| *MCC* | 1.17E-01 | NI | NI |
| *MCM3AP* | 5.31E-01 | NI | NI |
| *MDM2* | NI | NI | 1.72E-02 |
| *MED6* | NI | NI | 5.72E-01 |
| *MED8* | NI | NI | 8.50E-03 |
| *MEIG1* | 9.99E-01 | NI | NI |
| *MEIS1* | 9.76E-01 | NI | NI |
| *MEP1A* | 1.49E-02 | NI | NI |
| *MICA* | < 1.00E-06 | NI | NI |
| *MICB* | < 1.00E-06 | NI | NI |
| *MLC1* | NI | 5.48E-01 | NI |
| *MLLT6* | < 1.00E-06 | NI | NI |
| *MME* | 9.17E-01 | NI | NI |
| *MMP2* | 9.62E-01 | NI | NI |
| *MRGPRX2* | NI | 6.02E-01 | NI |
| *MRPL10* | 4.70E-01 | NI | NI |
| *MRPL38* | 4.26E-01 | NI | NI |
| *MRPL44* | 8.16E-01 | NI | NI |
| *MRPS18A* | 2.96E-01 | NI | NI |
| *MRPS22* | 7.91E-01 | NI | NI |
| *MVK* | 3.15E-01 | NI | NI |
| *MYBPC3* | 3.10E-05 | NI | NI |
| *MYC* | NI | NI | 1.64E-01 |
| *MYH7B* | < 1.00E-06 | NI | NI |
| *MYH9* | 1.85E-02 | NI | NI |
| *MYO18B* | 5.46E-01 | NI | NI |
| *MYOM1* | 1.98E-01 | NI | NI |
| *N4BP2L2* | NI | NI | < 1.00E-06 |
| *NAF1* | 9.57E-01 | NI | NI |
| *NAPA* | NI | 1.10E-02 | NI |
| *NAPSA* | 9.22E-01 | NI | NI |
| *NAT1* | 5.52E-01 | NI | NI |
| *NCAPG* | < 1.00E-06 | NI | NI |
| *NCOA1* | 2.00E-06 | NI | NI |
| *NCOA6* | < 1.00E-06 | NI | NI |
| *NCOR2* | 2.80E-05 | NI | NI |
| *NDEL1* | 5.75E-01 | NI | NI |
| *NECAB3* | < 1.00E-06 | NI | NI |
| *NEU1* | < 1.00E-06 | NI | < 1.00E-06 |
| *NFATC4* | < 1.00E-06 | NI | NI |
| *NFIC* | < 1.00E-06 | NI | 5.00E-06 |
| *NFKB1* | 1.01E-02 | NI | NI |
| *NFKBIL1* | < 1.00E-06 | NI | NI |
| *NFS1* | < 1.00E-06 | NI | < 1.00E-06 |
| *NHEJ1* | 6.92E-04 | NI | NI |
| *NME7* | NI | 1.78E-01 | NI |
| *NMU* | 2.49E-02 | NI | NI |
| *NOTCH4* | < 1.00E-06 | NI | NI |
| *NPM1* | 1.39E-04 | NI | 4.16E-04 |
| *NPPC* | < 1.00E-06 | NI | NI |
| *NPR3* | < 1.00E-06 | NI | NI |
| *NR4A1* | 4.42E-01 | NI | NI |
| *NRF1* | 7.90E-01 | NI | 4.92E-01 |
| *NSF* | 9.37E-03 | NI | NI |
| *NUCB1* | 9.51E-01 | NI | NI |
| *NUDT3* | 2.42E-04 | 5.97E-02 | NI |
| *NUP153* | NI | 1.50E-05 | NI |
| *NUP205* | 7.39E-04 | NI | NI |
| *OGG1* | 9.62E-01 | NI | NI |
| *OGN* | 1.30E-05 | NI | NI |
| *OLFML2A* | 1.79E-01 | NI | NI |
| *ORM1* | 3.53E-02 | NI | NI |
| *OSGEP* | 1.51E-02 | NI | NI |
| *OTUD4* | 1.50E-05 | NI | NI |
| *P4HA2* | < 1.00E-06 | NI | < 1.00E-06 |
| *PACSIN1* | 3.00E-05 | NI | NI |
| *PADI2* | < 1.00E-06 | NI | NI |
| *PAICS* | NI | 2.17E-03 | NI |
| *PAM* | 1.66E-02 | NI | NI |
| *PAXIP1* | NI | NI | 9.80E-01 |
| *PBX2* | < 1.00E-06 | NI | NI |
| *PCBP4* | 4.15E-01 | NI | NI |
| *PDIA4* | 8.12E-03 | NI | NI |
| *PDILT* | 3.54E-01 | NI | NI |
| *PDLIM4* | < 1.00E-06 | NI | < 1.00E-06 |
| *PDS5B* | < 1.00E-06 | NI | < 1.00E-06 |
| *PEX1* | < 1.00E-06 | NI | < 1.00E-06 |
| *PFDN5* | 6.58E-03 | NI | NI |
| *PGF* | 8.30E-03 | NI | NI |
| *PGM2L1* | 8.37E-03 | NI | NI |
| *PHB* | 1.85E-03 | NI | NI |
| *PHF20* | < 1.00E-06 | NI | < 1.00E-06 |
| *PHF3* | 1.22E-01 | NI | NI |
| *PIGU* | < 1.00E-06 | NI | NI |
| *PIK3CA* | NI | NI | 9.04E-01 |
| *PIK3R3* | 5.33E-04 | NI | NI |
| *PLAA* | 3.36E-01 | NI | NI |
| *PLEKHJ1* | < 1.00E-06 | NI | NI |
| *PLG* | 3.32E-01 | NI | NI |
| *PLK1* | NI | 3.30E-02 | NI |
| *PML* | 3.56E-03 | NI | 2.76E-02 |
| *POLR2H* | NI | 2.28E-02 | NI |
| *POLR3B* | NI | 2.32E-02 | NI |
| *POM121C* | 8.03E-01 | NI | NI |
| *POMC* | 4.34E-02 | NI | NI |
| *POU5F1* | 3.00E-06 | NI | 2.84E-04 |
| *PPAP2A* | NI | 1.05E-02 | NI |
| *PPARD* | < 1.00E-06 | NI | NI |
| *PPAT* | NI | 2.29E-03 | NI |
| *PPIA* | NI | 8.96E-03 | NI |
| *PPM1J* | 9.13E-01 | NI | NI |
| *PPP2R3C* | NI | 1.92E-04 | NI |
| *PPP2R5D* | 4.13E-03 | NI | NI |
| *PPP3CA* | 8.99E-01 | NI | NI |
| *PPT2* | < 1.00E-06 | NI | NI |
| *PPWD1* | 7.03E-01 | NI | NI |
| *PRDX6* | 5.55E-01 | NI | NI |
| *PRKAA1* | NI | NI | 3.07E-01 |
| *PRKAG3* | 1.45E-01 | NI | NI |
| *PRKCA* | NI | NI | 6.85E-03 |
| *PRKCZ* | 7.00E-05 | NI | NI |
| *PRKD3* | NI | 8.53E-03 | NI |
| *PROCR* | < 1.00E-06 | NI | < 1.00E-06 |
| *PRPSAP2* | 6.00E-01 | NI | NI |
| *PRTN3* | NI | NI | 2.15E-01 |
| *PRX* | 2.93E-01 | NI | NI |
| *PSMA6* | 2.79E-03 | NI | NI |
| *PSMB3* | 2.00E-06 | NI | NI |
| *PSMC3* | 8.00E-06 | NI | NI |
| *PSMC5* | < 1.00E-06 | NI | < 1.00E-06 |
| *PSTPIP1* | 3.09E-03 | NI | NI |
| *PTK2* | NI | 1.61E-01 | NI |
| *PTMA* | NI | 4.88E-01 | NI |
| *PTN* | NI | 1.52E-02 | NI |
| *PTP4A3* | NI | 1.33E-02 | NI |
| *PTPN1* | 1.61E-03 | NI | NI |
| *PTPN6* | 1.21E-01 | NI | NI |
| *PTPRC* | 9.33E-01 | NI | NI |
| *PTPRJ* | 2.90E-05 | NI | NI |
| *PXMP4* | < 1.00E-06 | NI | NI |
| *RAB1B* | 2.10E-05 | NI | NI |
| *RAB23* | NI | 1.14E-02 | NI |
| *RAPSN* | 8.00E-06 | NI | NI |
| *RASSF1* | 8.25E-03 | NI | NI |
| *RBL1* | 3.26E-04 | NI | NI |
| *RBM11* | 9.78E-01 | NI | NI |
| *RBM12* | < 1.00E-06 | 6.40E-03 | < 1.00E-06 |
| *RBM39* | < 1.00E-06 | 1.40E-02 | < 1.00E-06 |
| *RBM6* | 5.62E-01 | NI | NI |
| *RBPMS2* | 8.34E-01 | NI | NI |
| *RBX1* | 1.56E-01 | NI | NI |
| *REL* | 2.46E-02 | NI | NI |
| *RELA* | 6.06E-01 | NI | NI |
| *RFPL4B* | 5.70E-02 | NI | NI |
| *RGS4* | 5.68E-01 | NI | NI |
| *RHOA* | 5.88E-01 | NI | NI |
| *RHPN1* | 5.99E-01 | NI | NI |
| *RIPK1* | 6.70E-01 | NI | NI |
| *RIPK3* | 1.00E-06 | NI | NI |
| *RND3* | 3.46E-01 | NI | NI |
| *RNF144B* | NI | 1.81E-02 | NI |
| *RNF2* | 5.59E-01 | NI | NI |
| *RNF25* | < 1.00E-06 | NI | NI |
| *RNF5* | < 1.00E-06 | NI | NI |
| *ROPN1* | 3.18E-02 | NI | NI |
| *RPL10A* | < 1.00E-06 | NI | NI |
| *RPL13A* | 6.27E-01 | NI | NI |
| *RPL21* | 1.97E-02 | NI | NI |
| *RPL23* | 5.72E-04 | NI | NI |
| *RPL24* | 4.22E-01 | NI | NI |
| *RPL27* | 3.78E-01 | NI | NI |
| *RPL4* | 5.03E-01 | NI | NI |
| *RPL5* | 2.20E-05 | NI | NI |
| *RPLP2* | NI | 3.14E-03 | NI |
| *RPS2* | 2.82E-03 | NI | NI |
| *RPS27A* | 9.48E-01 | NI | NI |
| *RPS27L* | 2.17E-04 | NI | NI |
| *RUSC1* | 6.53E-01 | NI | NI |
| *RUVBL1* | 5.76E-01 | NI | NI |
| *RYBP* | 1.00E-06 | NI | < 1.00E-06 |
| *SALL1* | 2.71E-01 | NI | NI |
| *SCAND1* | < 1.00E-06 | NI | < 1.00E-06 |
| *SCG5* | 2.15E-01 | NI | NI |
| *SCMH1* | < 1.00E-06 | NI | < 1.00E-06 |
| *SCUBE3* | < 1.00E-06 | NI | NI |
| *SDCBP* | 7.31E-02 | NI | NI |
| *SDCBP2* | 9.12E-01 | NI | NI |
| *SDHB* | < 1.00E-06 | NI | NI |
| *SEC63* | NI | 2.14E-02 | NI |
| *SEL1L* | NI | 4.69E-01 | NI |
| *SENP1* | NI | 8.38E-04 | NI |
| *SEPT2* | < 1.00E-06 | NI | NI |
| *SERPING1* | 5.39E-02 | NI | NI |
| *SETMAR* | 1.66E-01 | NI | NI |
| *SF3A2* | < 1.00E-06 | NI | 1.52E-04 |
| *SF3B3* | NI | NI | 3.56E-02 |
| *SF3B4* | 1.40E-05 | NI | NI |
| *SFXN3* | 2.75E-04 | NI | NI |
| *SFXN4* | NI | 3.20E-03 | NI |
| *SGCA* | 2.09E-01 | NI | NI |
| *SGCD* | 7.23E-01 | NI | NI |
| *SH3RF2* | NI | 1.44E-02 | NI |
| *SHB* | 2.44E-01 | NI | NI |
| *SIGLEC10* | 7.51E-01 | NI | NI |
| *SIM1* | 6.07E-01 | NI | NI |
| *SIN3A* | 5.98E-03 | NI | NI |
| *SIRT1* | NI | NI | 1.75E-03 |
| *SIVA1* | NI | NI | 1.37E-02 |
| *SIX6* | < 1.00E-06 | NI | NI |
| *SKIV2L* | < 1.00E-06 | NI | NI |
| *SKP1* | 1.05E-01 | NI | NI |
| *SKP2* | 1.51E-02 | NI | NI |
| *SLC22A4* | NI | NI | < 1.00E-06 |
| *SLC22A5* | < 1.00E-06 | NI | < 1.00E-06 |
| *SLC25A22* | NI | 3.26E-03 | NI |
| *SLC9A3R2* | NI | NI | 1.19E-02 |
| *SLFNL1* | < 1.00E-06 | NI | < 1.00E-06 |
| *SLIT2* | 8.46E-01 | NI | NI |
| *SMAD3* | 7.18E-03 | NI | 5.06E-04 |
| *SMAD7* | 3.07E-03 | NI | NI |
| *SMARCA2* | NI | 1.04E-01 | NI |
| *SMARCD2* | < 1.00E-06 | NI | NI |
| *SMARCD3* | NI | 1.82E-02 | NI |
| *SMPD3* | 2.34E-01 | NI | NI |
| *SMURF1* | 1.90E-02 | NI | NI |
| *SNIP1* | NI | 1.32E-03 | NI |
| *SNTA1* | 1.95E-04 | NI | 9.70E-03 |
| *SNX17* | 8.44E-03 | NI | NI |
| *SOX30* | 1.48E-01 | 1.97E-01 | NI |
| *SP1* | 8.02E-03 | NI | NI |
| *SP2* | 2.00E-06 | NI | NI |
| *SPAG16* | 8.72E-01 | NI | NI |
| *SPAG4* | NI | NI | < 1.00E-06 |
| *SPAG6* | 4.75E-02 | NI | NI |
| *SPARC* | 4.85E-01 | NI | NI |
| *SPCS2* | 3.10E-05 | NI | NI |
| *SPI1* | 1.10E-05 | NI | NI |
| *SPRED1* | 8.22E-03 | NI | NI |
| *SPTBN1* | 8.37E-03 | NI | NI |
| *SRC* | 1.90E-01 | NI | NI |
| *SRF* | 1.56E-03 | NI | NI |
| *SSTR3* | NI | 9.85E-01 | NI |
| *ST6GALNAC4* | NI | 9.90E-05 | NI |
| *STAT1* | 7.34E-04 | NI | NI |
| *STIM1* | 3.99E-01 | NI | NI |
| *STK25* | 6.00E-06 | NI | NI |
| *STMN1* | 1.00E+00 | NI | NI |
| *STUB1* | NI | NI | 4.85E-04 |
| *SUMO1* | 1.08E-04 | NI | 5.19E-01 |
| *SUMO2* | 3.76E-01 | 3.04E-01 | NI |
| *SUPT3H* | < 1.00E-06 | NI | < 1.00E-06 |
| *SUPT4H1* | 2.09E-01 | NI | NI |
| *SYNCRIP* | NI | NI | 2.82E-01 |
| *SYT1* | 3.89E-01 | NI | NI |
| *TACC3* | 7.00E-06 | NI | NI |
| *TAF10* | NI | 6.98E-01 | NI |
| *TAF11* | 2.45E-03 | NI | NI |
| *TAF7* | 5.11E-02 | 1.79E-02 | NI |
| *TALDO1* | NI | 2.88E-03 | NI |
| *TAPBP* | NI | NI | 7.19E-01 |
| *TARBP2* | 3.06E-01 | NI | NI |
| *TARDBP* | 9.59E-04 | NI | NI |
| *TBC1D4* | NI | 1.17E-03 | NI |
| *TBX2* | < 1.00E-06 | NI | < 1.00E-06 |
| *TCEA1* | 1.22E-01 | NI | NI |
| *TCF19* | 3.00E-06 | NI | NI |
| *TCOF1* | NI | 4.59E-01 | NI |
| *TEAD1* | < 1.00E-06 | NI | NI |
| *TEAD3* | < 1.00E-06 | NI | NI |
| *TEK* | 7.51E-01 | NI | NI |
| *TFIP11* | 7.98E-01 | NI | NI |
| *TFR2* | NI | 3.84E-01 | NI |
| *TGFB2* | < 1.00E-06 | 1.36E-02 | NI |
| *TGFBR1* | 1.06E-02 | NI | NI |
| *TLX1* | 5.65E-01 | NI | NI |
| *TLX2* | 4.79E-03 | NI | NI |
| *TMED10* | 2.05E-04 | NI | NI |
| *TMEM129* | NI | 7.80E-02 | NI |
| *TMEM14A* | NI | 1.12E-02 | NI |
| *TMEM173* | 8.52E-03 | NI | NI |
| *TNF* | < 1.00E-06 | NI | 1.43E-04 |
| *TNFRSF14* | 2.73E-01 | NI | NI |
| *TNFRSF1A* | 4.89E-01 | NI | NI |
| *TNFSF4* | 5.66E-01 | NI | NI |
| *TNNI3K* | NI | 7.88E-01 | NI |
| *TNXB* | < 1.00E-06 | NI | NI |
| *TOB1* | 7.58E-02 | NI | NI |
| *TP53* | 7.70E-03 | NI | 2.47E-01 |
| *TP53I3* | < 1.00E-06 | NI | NI |
| *TPCN2* | NI | 8.83E-01 | NI |
| *TRAF2* | 7.33E-02 | NI | NI |
| *TRAF3IP2* | 2.83E-02 | NI | NI |
| *TRAF4* | 7.82E-01 | NI | NI |
| *TRAF7* | 1.20E-05 | NI | NI |
| *TRIM13* | 2.00E-06 | NI | NI |
| *TRIM27* | 5.20E-05 | NI | NI |
| *TRIM54* | 2.10E-02 | NI | NI |
| *TRIM63* | 3.22E-03 | NI | NI |
| *TRPC1* | 4.81E-01 | NI | NI |
| *TRPC4AP* | 2.30E-05 | NI | NI |
| *TSC2* | 8.00E-06 | NI | NI |
| *TSC22D1* | 8.16E-03 | 2.25E-02 | NI |
| *TTF2* | 7.56E-01 | NI | NI |
| *TUBA1A* | 9.88E-03 | NI | NI |
| *TUBA1C* | 3.35E-01 | NI | NI |
| *TUBB* | 1.23E-02 | NI | NI |
| *TUBG1* | 4.49E-03 | NI | NI |
| *TUFT1* | 2.75E-02 | NI | NI |
| *TXNRD2* | NI | 1.39E-02 | NI |
| *UBC* | 1.22E-01 | 8.25E-01 | 7.04E-01 |
| *UBD* | 1.96E-02 | NI | NI |
| *UBE2D2* | 1.63E-02 | NI | NI |
| *UBE2D3* | 2.38E-01 | NI | NI |
| *UBE2E1* | 9.90E-01 | NI | NI |
| *UBE2G2* | 5.67E-01 | NI | NI |
| *UBE2I* | NI | 6.48E-02 | NI |
| *UBE2Z* | 1.21E-03 | NI | NI |
| *UBQLN4* | NI | NI | 3.36E-02 |
| *UBR5* | NI | 1.63E-02 | NI |
| *UIMC1* | < 1.00E-06 | NI | NI |
| *UMPS* | 5.77E-02 | NI | NI |
| *USHBP1* | 7.69E-01 | NI | NI |
| *USP15* | 1.91E-03 | NI | NI |
| *USP20* | NI | 2.17E-03 | NI |
| *USP47* | 2.96E-01 | NI | NI |
| *USP7* | 2.79E-02 | NI | NI |
| *UTP15* | NI | 1.17E-02 | NI |
| *VAMP2* | 3.05E-03 | NI | NI |
| *VAMP8* | 5.28E-01 | NI | NI |
| *VARS* | NI | NI | 1.48E-04 |
| *VAV3* | 4.81E-01 | NI | NI |
| *VDAC2* | 7.69E-03 | NI | NI |
| *VEGFA* | 3.49E-02 | NI | NI |
| *VLDLR* | 9.17E-01 | NI | NI |
| *VPRBP* | NI | 1.97E-02 | NI |
| *VTI1B* | 7.25E-02 | NI | NI |
| *VTN* | NI | 1.27E-01 | NI |
| *WDR37* | 5.67E-01 | NI | NI |
| *WDR48* | NI | 2.66E-02 | NI |
| *WDR89* | 6.78E-01 | NI | NI |
| *WNT5B* | 5.95E-01 | NI | NI |
| *XPNPEP1* | NI | 2.32E-02 | NI |
| *XRCC1* | 8.18E-03 | NI | NI |
| *XRCC4* | 1.34E-01 | NI | NI |
| *XRN2* | 8.38E-03 | NI | NI |
| *YIF1B* | 6.78E-01 | NI | NI |
| *YWHAH* | 8.03E-01 | NI | NI |
| *YWHAZ* | NI | 5.90E-01 | NI |
| *ZBTB32* | 2.28E-03 | NI | NI |
| *ZDHHC17* | 8.84E-01 | NI | 2.32E-01 |
| *ZDHHC4* | 1.53E-01 | NI | NI |
| *ZNF195* | 2.61E-01 | NI | NI |
| *ZNF211* | 1.77E-01 | NI | NI |
| *ZNF239* | 7.54E-01 | NI | NI |
| *ZNF311* | < 1.00E-06 | NI | NI |
| *ZNF341* | < 1.00E-06 | NI | NI |
| *ZNF451* | NI | 1.37E-02 | NI |
| *ZNF462* | < 1.00E-06 | NI | NI |
| *ZNF593* | 2.70E-05 | NI | NI |
| *ZNF599* | 9.16E-01 | NI | NI |
| *ZNF638* | 3.73E-04 | NI | NI |
| *ZNF76* | < 1.00E-06 | NI | NI |
| *ZNFX1* | 7.00E-06 | NI | NI |
| *ZNRD1* | 4.89E-03 | NI | NI |
