# Supplementary material for: Evidence of Polygenic Adaptation in the Systems Genetics of Anthropometric Traits
Source: PLoS One. 2016 Aug 18;11(8):e0160654. doi: 10.1371/journal.pone.0160654 (PMC4990182; doi:10.1371/journal.pone.0160654)
Supplement: S6 Table — (DOCX) [file pone.0160654.s006.docx]

**S6 Table**: Significant long-distance genotypic LDs observed in Height-associated gene networks.

| **rsId1** | **chr1** | **loc1** | **rsId2** | **chr2** | **loc2** | **p.value** | **q.value** | **SNP1_p** | **SNP1_iHS** | **SNP2_p** | **SNP2_iHS** | **Gene1** | **Gene1_p** | **Gene2** | **Gene2_p** | **Height-related phenotype** |
| --- | --- | --- | --- | --- | --- | --- | --- | --- | --- | --- | --- | --- | --- | --- | --- | --- |
| rs3765895 | 1 | 10996837 | rs6453619 | 6 | 73800230 | < 1.00E-06 | < 5.00E-02 | 4.24E-02 | 1.71 | 1.79E-01 | 2.31 | *SRM* | 5.33E-04 | *KCNQ5* | 7.99E-01 | Distribution |
| rs213769 | 1 | 41387945 | rs4077176 | 9 | 94867017 | < 1.00E-06 | < 5.00E-02 | 9.39E-05 | 1.75 | 6.75E-01 | -1.76 | *SCMH1* | < 1.00E-06 | *FGD3* | 1.47E-01 | Distribution |
| rs2275426 | 1 | 46260139 | rs9614176 | 22 | 42253832 | < 1.00E-06 | < 5.00E-02 | 3.68E-03 | -1.52 | 1.25E-01 | -1.79 | *PIK3R3* | 5.33E-04 | *MPPED1* | 3.20E-02 | Distribution |
| rs9428015 | 1 | 89041928 | rs850319 | 3 | 188429536 | < 1.00E-06 | < 5.00E-02 | 1.61E-04 | 1.93 | 1.21E-01 | -2.13 | *PKN2* | 1.10E-05 | *MASP1* | 2.79E-01 | Distribution |
| rs3767569 | 1 | 91945959 | rs2844523 | 6 | 31476567 | < 1.00E-06 | < 5.00E-02 | 5.40E-01 | -1.50 | 8.20E-01 | -1.78 | *TGFBR3* | 4.16E-01 | *MICA* | 6.00E-06 | Extreme phenotype differences |
| rs1891592 | 1 | 149821127 | rs11704029 | 22 | 42546522 | < 1.00E-06 | < 5.00E-02 | 2.20E-02 | 2.50 | 5.89E-01 | -2.14 | *SNX27* | 1.46E-02 | *SULT4A1* | 3.22E-02 | Distribution |
| rs6691456 | 1 | 161357045 | rs6760896 | 2 | 232881167 | < 1.00E-06 | < 5.00E-02 | 3.63E-01 | 1.56 | 4.77E-03 | -1.52 | *RGS4* | 5.68E-01 | *DIS3L2* | < 1.00E-06 | Distribution |
| rs2670752 | 2 | 71427316 | rs2239749 | 22 | 35661251 | < 1.00E-06 | < 5.00E-02 | 7.10E-02 | 1.60 | 5.30E-01 | 2.29 | *ZNF638* | 3.99E-03 | *CSF2RB* | 9.24E-01 | Extreme phenotype differences |
| rs4851756 | 2 | 105300500 | rs568298 | 10 | 111645055 | < 1.00E-06 | < 5.00E-02 | 7.90E-01 | -1.58 | 3.40E-01 | -1.57 | *C2orf49* | 1.92E-02 | *XPNPEP1* | 2.32E-02 | Phenotypic variability |
| rs1017323 | 2 | 179196981 | rs8042108 | 15 | 87205884 | < 1.00E-06 | < 5.00E-02 | 1.20E-01 | -2.38 | 2.00E-02 | 2.39 | *TTN* | 7.70E-02 | *ACAN* | 3.97E-04 | Extreme phenotype differences |
| rs7424790 | 2 | 189610435 | rs653536 | 6 | 33768389 | < 1.00E-06 | < 5.00E-02 | 4.45E-02 | -1.50 | 8.94E-01 | -2.95 | *COL5A2* | 7.89E-02 | *IHPK3* | < 1.00E-06 | Distribution |
| rs7424790 | 2 | 189610435 | rs525623 | 6 | 33780581 | < 1.00E-06 | < 5.00E-02 | 4.45E-02 | -1.50 | 4.36E-01 | -2.58 | *COL5A2* | 7.89E-02 | *IHPK3* | < 1.00E-06 | Distribution |
| rs7424790 | 2 | 189610435 | rs649775 | 6 | 33792291 | < 1.00E-06 | < 5.00E-02 | 4.45E-02 | -1.50 | 5.98E-01 | -3.26 | *COL5A2* | 7.89E-02 | *C6orf125* | < 1.00E-06 | Distribution |
| rs11887698 | 2 | 191563119 | rs16915382 | 11 | 6535930 | < 1.00E-06 | < 5.00E-02 | 6.81E-01 | -1.61 | 8.88E-02 | 1.67 | *GLS* | 4.26E-04 | *ILK* | 1.05E-01 | Distribution |
| rs6740131 | 2 | 191597827 | rs3798465 | 6 | 73809830 | < 1.00E-06 | < 5.00E-02 | 3.43E-01 | -1.56 | 5.19E-01 | 2.76 | *STAT1* | 7.34E-04 | *KCNQ5* | 7.99E-01 | Distribution |
| rs941374 | 2 | 197019585 | rs11634989 | 15 | 36320860 | < 1.00E-06 | < 5.00E-02 | 5.29E-01 | -2.12 | 1.25E-01 | -1.51 | *HECW2* | 6.43E-01 | *SPRED1* | 8.22E-03 | Distribution |
| rs4241191 | 2 | 197190452 | rs2055566 | 2 | 241999610 | < 1.00E-06 | < 5.00E-02 | 4.90E-01 | 2.84 | 1.53E-01 | -2.11 | *HECW2* | 6.43E-01 | *FARP2* | < 1.00E-06 | Distribution |
| rs13002576 | 2 | 215909152 | rs2237142 | 6 | 15553778 | < 1.00E-06 | < 5.00E-02 | 8.50E-02 | 1.56 | 9.88E-01 | -1.76 | *FN1* | 4.85E-02 | *JARID2* | 1.00E-01 | Distribution |
| rs3770472 | 2 | 217266980 | rs11057560 | 12 | 123332538 | < 1.00E-06 | < 5.00E-02 | 1.71E-01 | 1.55 | 3.26E-06 | -1.66 | *IGFBP5* | 6.71E-01 | *FAM101A* | 4.00E-06 | Distribution |
| rs2582349 | 3 | 4368415 | rs11906160 | 20 | 33029416 | < 1.00E-06 | < 5.00E-02 | 4.42E-02 | 2.01 | 7.10E-05 | -1.85 | *SETMAR* | 1.66E-01 | *MYH7B* | < 1.00E-06 | Distribution |
| rs6442262 | 3 | 11603813 | rs11572177 | 10 | 96787260 | < 1.00E-06 | < 5.00E-02 | 2.80E-01 | 1.64 | 6.50E-01 | 1.52 | *ATG7* | 4.80E-04 | *CYP2C9* | 7.33E-01 | Extreme phenotype differences |
| rs11564445 | 3 | 41242756 | rs12107249 | 3 | 140539649 | < 1.00E-06 | < 5.00E-02 | 4.20E-02 | 1.91 | 2.32E-01 | 2.08 | *ULK4* | 2.75E-02 | *MRPS22* | 7.91E-01 | Distribution |
| rs11709183 | 3 | 109790360 | rs2271715 | 15 | 87250631 | < 1.00E-06 | < 5.00E-02 | 7.03E-01 | -2.36 | 5.42E-04 | -1.72 | *DZIP3* | 7.58E-02 | *ACAN* | 9.00E-06 | Distribution |
| rs2332682 | 3 | 125137323 | rs1990291 | 17 | 56729407 | < 1.00E-06 | < 5.00E-02 | 5.22E-02 | -2.28 | 3.66E-01 | 2.77 | *ROPN1* | 3.18E-02 | *BCAS3* | < 1.00E-06 | Distribution |
| rs586395 | 3 | 125884382 | rs6946304 | 7 | 148377157 | < 1.00E-06 | < 5.00E-02 | 6.30E-02 | 1.78 | 4.49E-01 | 1.98 | *KALRN* | 1.40E-02 | *PDIA4* | 8.12E-03 | Distribution |
| rs7650365 | 3 | 129316693 | rs3135050 | 6 | 29736416 | < 1.00E-06 | < 5.00E-02 | 3.77E-01 | -2.45 | 4.24E-02 | -1.74 | *SEC61A1* | 4.77E-01 | *MOG* | 1.15E-04 | Distribution |
| rs7650365 | 3 | 129316693 | rs3132679 | 6 | 30183822 | < 1.00E-06 | < 5.00E-02 | 3.77E-01 | -2.45 | 3.06E-02 | -2.43 | *SEC61A1* | 4.77E-01 | *RNF39* | 4.59E-03 | Distribution |
| rs6795618 | 3 | 173528466 | rs10761192 | 9 | 94879239 | < 1.00E-06 | < 5.00E-02 | 7.78E-03 | 1.73 | 8.67E-01 | 2.14 | *FNDC3B* | < 1.00E-06 | *FGD3* | 1.47E-01 | Distribution |
| rs6795618 | 3 | 173528466 | rs4843285 | 16 | 86557072 | < 1.00E-06 | < 5.00E-02 | 7.78E-03 | 1.73 | 3.15E-02 | 1.67 | *FNDC3B* | < 1.00E-06 | *CA5A* | 9.05E-03 | Distribution |
| rs4690055 | 4 | 2718461 | rs2304717 | 15 | 72113610 | < 1.00E-06 | < 5.00E-02 | 2.20E-01 | 2.03 | 3.00E-02 | 1.74 | *C4orf8* | 6.59E-01 | *GOLGA6* | 2.07E-02 | Extreme phenotype differences |
| rs6849536 | 4 | 20049574 | rs2280352 | 17 | 44186025 | < 1.00E-06 | < 5.00E-02 | 7.75E-01 | -1.68 | 7.19E-01 | 2.04 | *SLIT2* | 8.46E-01 | *TTLL6* | 6.53E-03 | Distribution |
| rs7690165 | 4 | 87971933 | rs6100232 | 20 | 56816338 | < 1.00E-06 | < 5.00E-02 | 8.10E-02 | 1.82 | 8.00E-03 | -1.67 | *PTPN13* | 1.25E-02 | *GNAS* | 3.22E-03 | Extreme phenotype differences |
| rs1912555 | 4 | 144641172 | rs3024745 | 13 | 112869599 | < 1.00E-06 | < 5.00E-02 | 1.43E-02 | 1.64 | 8.68E-02 | 1.79 | *GAB1* | 4.37E-04 | *F7* | 1.75E-02 | Distribution |
| rs33671 | 5 | 36188499 | rs720006 | 6 | 16755970 | < 1.00E-06 | < 5.00E-02 | 5.30E-03 | 2.05 | 3.30E-01 | 2.05 | *LMBRD2* | 1.26E-03 | *ATXN1* | 9.32E-01 | Extreme phenotype differences |
| rs17284253 | 5 | 82448823 | rs2240123 | 17 | 43508295 | < 1.00E-06 | < 5.00E-02 | 1.13E-02 | -1.70 | 5.63E-01 | -1.77 | *TMEM167A* | 1.49E-02 | *NFE2L1* | 7.00E-06 | Distribution |
| rs17327526 | 5 | 112748547 | rs2228233 | 14 | 23909005 | < 1.00E-06 | < 5.00E-02 | 9.35E-02 | -2.37 | 1.93E-05 | 2.27 | *MCC* | 1.17E-01 | *ADCY4* | < 1.00E-06 | Distribution |
| rs247008 | 5 | 131475003 | rs12765071 | 10 | 102825908 | < 1.00E-06 | < 5.00E-02 | 9.10E-06 | 1.70 | 8.70E-01 | 1.81 | *CSF2* | < 1.00E-06 | *PDZD7* | 2.00E-03 | Extreme phenotype differences |
| rs6880774 | 5 | 155742492 | rs362512 | 6 | 29638607 | < 1.00E-06 | < 5.00E-02 | 7.41E-01 | 1.70 | 1.86E-01 | 2.18 | *SGCD* | 7.23E-01 | *GABBR1* | 6.07E-03 | Distribution |
| rs2077681 | 6 | 3031099 | rs924079 | 12 | 115738362 | < 1.00E-06 | < 5.00E-02 | 7.53E-01 | 2.00 | 1.06E-01 | -1.88 | *BPHL* | 5.71E-01 | *HRK* | 3.55E-03 | Distribution |
| rs2071303 | 6 | 26199315 | rs3807645 | 7 | 77668027 | < 1.00E-06 | < 5.00E-02 | 1.42E-02 | 1.84 | 4.32E-01 | -2.03 | *HIST1H1C* | < 1.00E-06 | *MAGI2* | 9.47E-01 | Distribution |
| rs3132390 | 6 | 28940767 | rs1860413 | 17 | 56869243 | < 1.00E-06 | < 5.00E-02 | 4.90E-02 | 2.25 | 3.34E-03 | 1.86 | *TRIM27* | 5.20E-05 | *BCAS3* | < 1.00E-06 | Distribution |
| rs3130845 | 6 | 29031346 | rs1860413 | 17 | 56869243 | < 1.00E-06 | < 5.00E-02 | 4.85E-02 | -1.92 | 3.34E-03 | 1.86 | *ZNF311* | < 1.00E-06 | *BCAS3* | < 1.00E-06 | Distribution |
| rs9257877 | 6 | 29584487 | rs17679445 | 17 | 43377064 | < 1.00E-06 | < 5.00E-02 | 2.40E-02 | -1.76 | 9.00E-04 | -2.64 | *OR2H1* | 8.68E-03 | *PNPO* | 1.00E-06 | Distribution |
| rs12530421 | 6 | 29657422 | rs12705836 | 7 | 78385138 | < 1.00E-06 | < 5.00E-02 | 5.42E-03 | -1.77 | 6.87E-02 | -1.56 | *GABBR1* | 6.07E-03 | *MAGI2* | 9.47E-01 | Distribution |
| rs9366752 | 6 | 30132656 | rs3828758 | 6 | 43063831 | < 1.00E-06 | < 5.00E-02 | 9.35E-02 | -2.29 | 1.11E-03 | -1.54 | *RNF39* | 4.59E-03 | *MEA1* | 2.04E-03 | Distribution |
| rs1042147 | 6 | 31191135 | rs137803 | 22 | 42418082 | < 1.00E-06 | < 5.00E-02 | 1.63E-02 | 1.51 | 1.47E-01 | -1.75 | *C6orf15* | < 1.00E-06 | *EFCAB6* | 6.49E-02 | Distribution |
| rs1042147 | 6 | 31191135 | rs137803 | 22 | 42418082 | < 1.00E-06 | < 5.00E-02 | 1.50E-03 | 1.51 | 3.00E-01 | -1.75 | *CDSN* | 2.53E-04 | *EFCAB6* | 1.59E-01 | Extreme phenotype differences |
| rs2233952 | 6 | 31213870 | rs9656005 | 7 | 92046282 | < 1.00E-06 | < 5.00E-02 | 2.11E-02 | -2.00 | 5.93E-10 | -1.83 | *C6orf15* | < 1.00E-06 | *CDK6* | < 1.00E-06 | Distribution |
| rs9266380 | 6 | 31442456 | rs7947357 | 11 | 57170963 | < 1.00E-06 | < 5.00E-02 | 2.30E-01 | -2.47 | 5.38E-02 | 1.56 | *HLA-B* | < 1.00E-06 | *SERPING1* | 5.39E-02 | Distribution |
| rs9266380 | 6 | 31442456 | rs7947357 | 11 | 57170963 | < 1.00E-06 | < 5.00E-02 | 6.00E-01 | -2.47 | 2.40E-01 | 1.56 | *MICA* | 6.00E-06 | *ZDHHC5* | 2.23E-01 | Extreme phenotype differences |
| rs9266669 | 6 | 31456056 | rs4657 | 9 | 96888911 | < 1.00E-06 | < 5.00E-02 | 3.58E-03 | -4.08 | 6.32E-01 | 1.97 | *MICA* | < 1.00E-06 | *C9orf3* | 8.50E-05 | Distribution |
| rs2844538 | 6 | 31456858 | rs5750250 | 22 | 35038429 | < 1.00E-06 | < 5.00E-02 | 6.86E-01 | 1.79 | 4.02E-01 | 2.36 | *MICA* | < 1.00E-06 | *APOL1* | 1.49E-02 | Distribution |
| rs9266773 | 6 | 31460327 | rs7947357 | 11 | 57170963 | < 1.00E-06 | < 5.00E-02 | 3.67E-01 | -3.48 | 5.38E-02 | 1.56 | *HLA-B* | < 1.00E-06 | *SERPING1* | 5.39E-02 | Distribution |
| rs2524276 | 6 | 31516244 | rs2298694 | 21 | 46474790 | < 1.00E-06 | < 5.00E-02 | 1.11E-01 | -2.34 | 7.89E-01 | -1.61 | *HCP5* | < 1.00E-06 | *C21orf56* | 1.00E-02 | Distribution |
| rs3093995 | 6 | 31596883 | rs9472468 | 6 | 45434092 | < 1.00E-06 | < 5.00E-02 | 6.01E-09 | -2.11 | 3.69E-05 | 1.70 | *MICB* | < 1.00E-06 | *SUPT3H* | < 1.00E-06 | Distribution |
| rs3132935 | 6 | 32279053 | rs4535796 | 9 | 94721209 | < 1.00E-06 | < 5.00E-02 | 8.69E-02 | -1.61 | 3.25E-01 | -1.64 | *PBX2* | < 1.00E-06 | *ZNF484* | 6.03E-04 | Distribution |
| rs377763 | 6 | 32307122 | rs11772708 | 7 | 134915277 | < 1.00E-06 | < 5.00E-02 | 7.96E-04 | -1.70 | 7.94E-05 | -1.75 | *NOTCH4* | < 1.00E-06 | *NUP205* | 7.39E-04 | Distribution |
| rs7753949 | 6 | 34990372 | rs937793 | 15 | 88854587 | < 1.00E-06 | < 5.00E-02 | 5.57E-04 | -2.25 | 1.34E-01 | 1.95 | *ANKS1A* | 2.50E-04 | *IQGAP1* | 7.92E-02 | Distribution |
| rs12207462 | 6 | 82512770 | rs926777 | 6 | 152346740 | < 1.00E-06 | < 5.00E-02 | 2.00E-01 | -1.79 | 1.00E-01 | 1.73 | *FAM46A* | 7.25E-01 | *ESR1* | 2.91E-04 | Extreme phenotype differences |
| rs1268167 | 6 | 109114876 | rs13285411 | 9 | 130037689 | < 1.00E-06 | < 5.00E-02 | 1.80E-02 | 1.94 | 4.80E-01 | 2.50 | *FOXO3* | 5.45E-02 | *CIZ1* | 8.89E-02 | Extreme phenotype differences |
| rs6941014 | 6 | 112025801 | rs11783925 | 8 | 54933413 | < 1.00E-06 | < 5.00E-02 | 3.23E-02 | 1.67 | 5.40E-01 | -1.67 | *TRAF3IP2* | 2.83E-02 | *RGS20* | 7.05E-01 | Distribution |
| rs798548 | 7 | 2727461 | rs2414407 | 15 | 53343520 | < 1.00E-06 | < 5.00E-02 | 1.50E-16 | 1.78 | 1.20E-01 | -1.91 | *GNA12* | < 1.00E-06 | *RAB27A* | 7.09E-01 | Extreme phenotype differences |
| rs1636264 | 7 | 2831112 | rs10242235 | 7 | 78310372 | < 1.00E-06 | < 5.00E-02 | 2.31E-22 | 1.66 | 9.68E-01 | -2.03 | *GNA12* | < 1.00E-06 | *MAGI2* | 9.47E-01 | Distribution |
| rs1636264 | 7 | 2831112 | rs2414407 | 15 | 53343520 | < 1.00E-06 | < 5.00E-02 | 7.40E-15 | 1.66 | 1.20E-01 | -1.91 | *GNA12* | < 1.00E-06 | *RAB27A* | 7.09E-01 | Extreme phenotype differences |
| rs17169194 | 7 | 7647222 | rs6019483 | 20 | 46929063 | < 1.00E-06 | < 5.00E-02 | 8.30E-01 | -1.66 | 6.10E-02 | 2.24 | *RPA3* | 5.90E-02 | *ARFGEF2* | 6.39E-04 | Extreme phenotype differences |
| rs4141318 | 7 | 11486631 | rs2642580 | 10 | 71204136 | < 1.00E-06 | < 5.00E-02 | 2.40E-02 | 2.10 | 3.80E-04 | 1.56 | *THSD7A* | 8.45E-02 | *COL13A1* | 2.78E-03 | Extreme phenotype differences |
| rs6960116 | 7 | 11744949 | rs11907811 | 20 | 33891492 | < 1.00E-06 | < 5.00E-02 | 8.80E-01 | 1.56 | 2.40E-04 | 2.05 | *THSD7A* | 8.45E-02 | *PHF20* | < 1.00E-06 | Extreme phenotype differences |
| rs6960116 | 7 | 11744949 | rs3829829 | 20 | 34000574 | < 1.00E-06 | < 5.00E-02 | 8.80E-01 | 1.56 | 3.20E-03 | 1.61 | *THSD7A* | 8.45E-02 | *PHF20* | < 1.00E-06 | Extreme phenotype differences |
| rs757864 | 7 | 77509747 | rs698468 | 9 | 108849791 | < 1.00E-06 | < 5.00E-02 | 4.45E-01 | -1.70 | 9.47E-01 | 2.09 | *MAGI2* | 9.47E-01 | *ZNF462* | < 1.00E-06 | Distribution |
| rs3779289 | 7 | 77623527 | rs9895661 | 17 | 56811371 | < 1.00E-06 | < 5.00E-02 | 8.11E-01 | -2.11 | 2.43E-03 | 2.53 | *MAGI2* | 9.47E-01 | *BCAS3* | < 1.00E-06 | Distribution |
| rs3807645 | 7 | 77668027 | rs9895661 | 17 | 56811371 | < 1.00E-06 | < 5.00E-02 | 4.32E-01 | -2.03 | 2.43E-03 | 2.53 | *MAGI2* | 9.47E-01 | *BCAS3* | < 1.00E-06 | Distribution |
| rs757624 | 7 | 148134310 | rs4362279 | 13 | 74895200 | < 1.00E-06 | < 5.00E-02 | 6.50E-01 | -1.53 | 2.40E-01 | 2.28 | *CUL1* | 3.86E-01 | *TBC1D4* | 1.17E-03 | Phenotypic variability |
| rs16911047 | 9 | 27123016 | rs2074977 | 19 | 3385028 | < 1.00E-06 | < 5.00E-02 | 6.66E-01 | -1.80 | 6.75E-07 | 2.00 | *TEK* | 7.51E-01 | *NFIC* | < 1.00E-06 | Distribution |
| rs6476362 | 9 | 32481060 | rs9568363 | 13 | 49429768 | < 1.00E-06 | < 5.00E-02 | 5.48E-01 | -1.55 | 3.41E-08 | 1.86 | *ACO1* | 7.75E-02 | *TRIM13* | 2.00E-06 | Distribution |
| rs296894 | 9 | 85788264 | rs2830113 | 21 | 26496353 | < 1.00E-06 | < 5.00E-02 | 5.30E-05 | -2.01 | 6.60E-01 | 2.38 | *RMI1* | 7.40E-05 | *APP* | 7.49E-02 | Extreme phenotype differences |
| rs4734 | 9 | 130058460 | rs2829946 | 21 | 26138228 | < 1.00E-06 | < 5.00E-02 | 5.53E-01 | 1.80 | 9.35E-02 | -1.65 | *GOLGA2* | 4.71E-03 | *APP* | 7.90E-02 | Distribution |
| rs7922341 | 10 | 14992798 | rs6507898 | 18 | 45126030 | < 1.00E-06 | < 5.00E-02 | 6.24E-01 | -1.50 | 1.03E-02 | -1.93 | *HSPA14* | 4.05E-01 | *DYM* | < 1.00E-06 | Distribution |
| rs4746935 | 10 | 71367032 | rs738703 | 20 | 33654113 | < 1.00E-06 | < 5.00E-02 | 5.90E-01 | 1.65 | 2.60E-04 | 1.67 | *COL13A1* | 2.78E-03 | *RBM12* | < 1.00E-06 | Extreme phenotype differences |
| rs10762368 | 10 | 71779488 | rs876383 | 15 | 72120466 | < 1.00E-06 | < 5.00E-02 | 8.95E-01 | 2.41 | 3.04E-05 | -2.05 | *LRRC20* | 6.19E-01 | *GOLGA6* | 1.06E-03 | Distribution |
| rs1858790 | 11 | 3322147 | rs10846674 | 12 | 123496685 | < 1.00E-06 | < 5.00E-02 | 2.29E-01 | 2.65 | 3.90E-01 | -1.65 | *ZNF195* | 2.61E-01 | *NCOR2* | 2.80E-05 | Distribution |
| rs16915415 | 11 | 6540504 | rs8067056 | 17 | 41439785 | < 1.00E-06 | < 5.00E-02 | 1.73E-01 | 1.57 | 1.05E-02 | 2.01 | *ILK* | 1.05E-01 | *KIAA1267* | 1.63E-03 | Distribution |
| rs10769312 | 11 | 48051345 | rs2847325 | 18 | 693442 | < 1.00E-06 | < 5.00E-02 | 7.90E-08 | 2.22 | 6.90E-01 | 1.60 | *PTPRJ* | 3.30E-05 | *TYMS* | 7.21E-01 | Extreme phenotype differences |
| rs2155548 | 11 | 128309999 | rs107068 | 19 | 40896530 | < 1.00E-06 | < 5.00E-02 | 5.43E-02 | 1.99 | 1.90E-03 | -1.76 | *KCNJ5* | 8.40E-02 | *ZBTB32* | 2.28E-03 | Distribution |
| rs1245775 | 12 | 78284931 | rs2584622 | 17 | 59263899 | < 1.00E-06 | < 5.00E-02 | 2.12E-01 | 2.50 | 5.13E-03 | -1.54 | *SYT1* | 3.89E-01 | *GH2* | < 1.00E-06 | Distribution |
| rs10861975 | 12 | 78318721 | rs2584622 | 17 | 59263899 | < 1.00E-06 | < 5.00E-02 | 2.09E-01 | -1.98 | 5.13E-03 | -1.54 | *SYT1* | 3.89E-01 | *GH2* | < 1.00E-06 | Distribution |
| rs2072511 | 12 | 94905363 | rs4942336 | 13 | 43984776 | < 1.00E-06 | < 5.00E-02 | 5.17E-01 | -2.01 | 8.10E-03 | 1.61 | *HAL* | 4.45E-01 | *TSC22D1* | 8.16E-03 | Distribution |
| rs17384892 | 13 | 49443403 | rs6019483 | 20 | 46929063 | < 1.00E-06 | < 5.00E-02 | 1.27E-01 | -1.66 | 2.76E-05 | 2.24 | *TRIM13* | 2.00E-06 | *ARFGEF2* | < 1.00E-06 | Distribution |
| rs9521926 | 13 | 110204397 | rs1003531 | 19 | 2157575 | < 1.00E-06 | < 5.00E-02 | 1.57E-01 | 1.91 | 1.41E-04 | -1.69 | *ING1* | 7.64E-02 | *DOT1L* | < 1.00E-06 | Distribution |
| rs7157967 | 14 | 101655966 | rs11629568 | 15 | 65269422 | < 1.00E-06 | < 5.00E-02 | 1.70E-01 | 2.43 | 1.60E-01 | -1.83 | *HSP90AA1* | 4.01E-01 | *SMAD3* | 5.06E-04 | Extreme phenotype differences |
| rs1190583 | 14 | 101679429 | rs11629568 | 15 | 65269422 | < 1.00E-06 | < 5.00E-02 | 1.30E-01 | 2.43 | 1.60E-01 | -1.83 | *HSP90AA1* | 4.01E-01 | *SMAD3* | 5.06E-04 | Extreme phenotype differences |
| rs4774476 | 15 | 61194443 | rs174764 | 22 | 28051161 | < 1.00E-06 | < 5.00E-02 | 2.14E-02 | -1.83 | 5.22E-02 | -1.98 | *LACTB* | 1.56E-04 | *RASL10A* | 4.31E-02 | Distribution |
| rs9907723 | 17 | 18699578 | rs6058312 | 20 | 33787898 | < 1.00E-06 | < 5.00E-02 | 6.43E-01 | 1.97 | 6.78E-11 | 2.63 | *FAM18B* | 2.08E-01 | *PHF20* | < 1.00E-06 | Distribution |
| rs4924944 | 17 | 18750727 | rs2425137 | 20 | 33808810 | < 1.00E-06 | < 5.00E-02 | 5.22E-01 | 1.92 | 2.05E-10 | 2.74 | *PRPSAP2* | 6.00E-01 | *PHF20* | < 1.00E-06 | Distribution |
| rs10512429 | 17 | 23493500 | rs2050729 | 20 | 33757823 | < 1.00E-06 | < 5.00E-02 | 7.20E-01 | -2.55 | 2.80E-05 | -1.83 | *NLK* | 5.84E-01 | *CPNE1* | < 1.00E-06 | Extreme phenotype differences |
| rs7221968 | 17 | 61848731 | rs12973409 | 19 | 2147963 | < 1.00E-06 | < 5.00E-02 | 6.50E-01 | 2.15 | 4.00E-04 | 1.63 | *PRKCA* | 6.85E-03 | *PLEKHJ1* | 7.80E-05 | Extreme phenotype differences |
