# Supplementary material for: Evidence of Polygenic Adaptation in the Systems Genetics of Anthropometric Traits
Source: PLoS One. 2016 Aug 18;11(8):e0160654. doi: 10.1371/journal.pone.0160654 (PMC4990182; doi:10.1371/journal.pone.0160654)
Supplement: S7 Table — NI: not included in top-10 PPI modules. (DOCX) [file pone.0160654.s007.docx]

**S7 Table**: Genes and their correspondence p values present in gene network associated with WC-related phenotypes. NI: not included in top-10 PPI modules.

| **Gene** | **WC-men** | **WC-women** |
| --- | --- | --- |
| *ABL1* | 3.85E-02 | NI |
| *ACTB* | 2.29E-01 | NI |
| *ACTBL2* | NI | 4.15E-03 |
| *ACTG2* | NI | 6.87E-01 |
| *ACTR3* | NI | 7.30E-03 |
| *ADCY3* | 7.93E-01 | NI |
| *ADSSL1* | 1.09E-01 | NI |
| *AKIRIN2* | 4.55E-03 | NI |
| *ALB* | 8.14E-02 | NI |
| *ANAPC13* | NI | 3.08E-03 |
| *APP* | NI | 9.22E-01 |
| *ARHGEF12* | 1.87E-03 | NI |
| *ARPC2* | NI | 6.89E-02 |
| *ASNSD1* | 1.71E-04 | NI |
| *ATF3* | 6.03E-02 | NI |
| *ATG12* | 3.82E-02 | NI |
| *ATP13A2* | 8.00E-06 | NI |
| *AZIN1* | 2.28E-02 | NI |
| *BCCIP* | 5.72E-03 | NI |
| *BIN1* | 8.64E-01 | NI |
| *BMI1* | 3.66E-03 | NI |
| *BUB1* | 1.02E-03 | NI |
| *C12orf45* | 1.01E-01 | NI |
| *C17orf82* | NI | 2.23E-04 |
| *C1orf105* | NI | 1.18E-03 |
| *C6orf136* | 7.02E-04 | NI |
| *CABLES1* | 1.10E-05 | NI |
| *CACNA1C* | 6.47E-03 | NI |
| *CAMK2G* | 2.72E-01 | NI |
| *CAPNS1* | 4.28E-03 | NI |
| *CAPSL* | NI | 8.93E-04 |
| *CASP8AP2* | 6.90E-03 | NI |
| *CCHCR1* | 8.33E-04 | NI |
| *CDC25A* | 4.41E-03 | NI |
| *CHEK2* | NI | 1.72E-03 |
| *CIITA* | NI | 3.66E-03 |
| *CLU* | 6.67E-04 | NI |
| *COBLL1* | NI | 2.80E-05 |
| *COL6A1* | NI | 4.58E-03 |
| *COPS2* | 1.17E-02 | NI |
| *COPS3* | 7.33E-01 | NI |
| *CRHR1* | NI | 4.72E-03 |
| *CRK* | 9.34E-02 | NI |
| *CROCC* | 2.70E-05 | NI |
| *CSE1L* | 4.23E-03 | NI |
| *CSF2* | 2.03E-03 | NI |
| *CSNK2B* | 1.16E-02 | NI |
| *CST4* | 8.93E-01 | NI |
| *CUL1* | 1.95E-02 | NI |
| *CYB5A* | 4.18E-01 | NI |
| *CYP17A1* | 8.72E-02 | NI |
| *CYP2A6* | 6.92E-01 | NI |
| *DBP* | 9.71E-01 | NI |
| *DDR1* | 6.20E-03 | NI |
| *DDX27* | 9.13E-03 | NI |
| *DIS3L2* | 9.51E-04 | NI |
| *DOCK3* | NI | 9.04E-04 |
| *EEF1A1* | 4.10E-01 | NI |
| *EHMT2* | 4.15E-03 | NI |
| *ELAVL1* | 2.28E-01 | NI |
| *ELMOD1* | NI | 3.46E-01 |
| *EZR* | 7.53E-02 | NI |
| *F2RL2* | 1.83E-03 | NI |
| *FARS2* | NI | 1.94E-01 |
| *GAB2* | 2.99E-03 | NI |
| *GHITM* | 6.64E-01 | NI |
| *GIGYF2* | NI | 4.53E-01 |
| *GMCL1* | 4.15E-03 | NI |
| *GNA12* | 2.80E-03 | 1.35E-02 |
| *GNAS* | NI | 2.05E-01 |
| *GRB2* | 1.86E-01 | 2.00E-03 |
| *GTF3C2* | NI | 3.24E-03 |
| *HDAC2* | NI | 1.80E-01 |
| *HEY2* | NI | 7.54E-04 |
| *HIBADH* | 8.78E-01 | NI |
| *HINT1* | 1.12E-02 | NI |
| *HIST1H1B* | 5.70E-03 | NI |
| *HK1* | NI | 8.20E-01 |
| *HLA-B* | 3.85E-04 | NI |
| *HMGA1* | NI | < 1.00E-06 |
| *HNRNPA0* | 2.32E-02 | NI |
| *HSP90AA1* | 3.05E-02 | NI |
| *HYAL1* | NI | 8.40E-05 |
| *IDE* | 4.93E-03 | NI |
| *IFRD2* | NI | 3.66E-04 |
| *IGF1* | 2.85E-01 | NI |
| *IGFBP3* | 3.65E-02 | NI |
| *IL7R* | NI | 1.07E-03 |
| *ITGA2* | NI | 1.70E-04 |
| *KANK2* | NI | 5.54E-01 |
| *KLF14* | NI | 4.04E-01 |
| *KLF15* | NI | 2.57E-01 |
| *KLHL25* | 6.38E-03 | NI |
| *KRT35* | 2.95E-02 | NI |
| *KRT86* | 1.26E-01 | NI |
| *LACTB* | 1.41E-01 | NI |
| *LMF1* | NI | 1.15E-04 |
| *LRP2BP* | 4.33E-01 | NI |
| *LYAR* | 2.88E-02 | NI |
| *MAPT* | NI | 4.87E-03 |
| *MDC1* | 1.73E-02 | NI |
| *MICA* | 1.20E-04 | NI |
| *MOCS2* | NI | 2.01E-04 |
| *MYH9* | NI | 1.27E-01 |
| *NAT6* | NI | 3.67E-04 |
| *NCL* | NI | 6.64E-02 |
| *NCOA1* | NI | 4.25E-02 |
| *NCOA6* | 9.45E-03 | NI |
| *NISCH* | NI | 1.94E-03 |
| *NOS3* | NI | 3.54E-02 |
| *NRD1* | NI | 2.22E-02 |
| *NRF1* | 4.95E-01 | 2.42E-01 |
| *NSD1* | 8.26E-03 | NI |
| *NUDCD2* | 9.60E-03 | NI |
| *NUP50* | NI | 9.68E-04 |
| *OSTF1* | 4.20E-01 | NI |
| *PADI2* | 3.80E-05 | NI |
| *PARP2* | 4.14E-02 | NI |
| *PCK1* | NI | 2.73E-02 |
| *PDIA3* | NI | 1.18E-02 |
| *POLL* | NI | 9.46E-01 |
| *POLR2C* | 6.97E-01 | NI |
| *POU5F1* | 1.34E-03 | NI |
| *PPIE* | NI | 8.18E-01 |
| *PRKCA* | NI | 1.86E-01 |
| *PSMB5* | 7.15E-03 | NI |
| *PSMC3* | 7.53E-03 | NI |
| *PSMD14* | 1.88E-02 | NI |
| *RAB7A* | 2.21E-01 | NI |
| *RARS* | NI | 1.80E-02 |
| *RASSF1* | NI | 5.90E-05 |
| *RB1* | NI | 5.01E-03 |
| *RBMS1* | 7.40E-03 | NI |
| *RIC8B* | NI | 2.08E-02 |
| *RIOK2* | 1.26E-02 | NI |
| *RIT1* | NI | 1.94E-03 |
| *RNF32* | NI | 1.60E-02 |
| *RPA1* | NI | 2.07E-01 |
| *RPAIN* | 1.37E-01 | NI |
| *RPS27A* | 4.53E-03 | NI |
| *RXFP3* | NI | 6.25E-01 |
| *RYBP* | 2.43E-03 | NI |
| *SDHB* | 3.00E-05 | NI |
| *SEC61A1* | NI | 1.34E-01 |
| *SF3B4* | NI | 1.89E-02 |
| *SHKBP1* | 3.36E-03 | NI |
| *SIRT1* | NI | 5.33E-01 |
| *SLBP* | 5.44E-01 | NI |
| *SLC16A4* | 4.95E-01 | NI |
| *SNRPB* | NI | 3.42E-03 |
| *SNX13* | NI | 1.77E-01 |
| *SORL1* | 1.73E-01 | NI |
| *SOX8* | NI | 1.62E-04 |
| *SREBF1* | NI | 4.33E-03 |
| *STAU1* | 4.61E-03 | NI |
| *STK32C* | NI | 9.24E-04 |
| *STOML3* | 7.83E-02 | NI |
| *STON2* | 1.86E-04 | NI |
| *SYNJ2* | 3.90E-02 | NI |
| *SYPL1* | NI | 1.08E-01 |
| *TBCB* | 1.05E-02 | NI |
| *TCEA1* | 4.10E-01 | NI |
| *TEAD1* | NI | 1.51E-02 |
| *TGFB2* | 8.40E-05 | NI |
| *THG1L* | NI | 1.57E-01 |
| *THRA* | 6.97E-01 | NI |
| *TMEM159* | 3.17E-01 | NI |
| *TNPO3* | NI | 1.88E-02 |
| *TP53* | 2.57E-01 | NI |
| *TP73* | 2.05E-02 | NI |
| *TRIM21* | 4.89E-02 | NI |
| *UBC* | 6.12E-01 | 3.83E-01 |
| *UBE2G1* | 2.52E-02 | NI |
| *UCN* | NI | 3.38E-03 |
| *UGT1A4* | 7.04E-02 | NI |
| *UIMC1* | 1.07E-03 | NI |
| *USP45* | NI | 2.79E-01 |
| *USP8* | 1.72E-01 | NI |
| *VTA1* | 2.44E-03 | NI |
| *WIPI1* | 7.69E-01 | NI |
| *XBP1* | NI | 1.76E-04 |
| *XPO1* | NI | 2.06E-02 |
| *YWHAE* | 3.09E-02 | NI |
| *YWHAG* | NI | 6.25E-03 |
| *ZFPM1* | 4.05E-01 | NI |
| *ZHX2* | 1.87E-02 | NI |
| *ZNF343* | NI | 1.61E-03 |
