# Supplementary material for: Evidence of Polygenic Adaptation in the Systems Genetics of Anthropometric Traits
Source: PLoS One. 2016 Aug 18;11(8):e0160654. doi: 10.1371/journal.pone.0160654 (PMC4990182; doi:10.1371/journal.pone.0160654)
Supplement: S8 Table — (DOCX) [file pone.0160654.s008.docx]

**S8 Table**: Significant long-distance genotypic LDs observed in WC-associated gene networks.

| **rsId1** | **chr1** | **loc1** | **rsId2** | **chr2** | **loc2** | **p.value** | **q.value** | **SNP1_p** | **SNP1_iHS** | **SNP2_p** | **SNP2_iHS** | **Gene1** | **Gene1_p** | **Gene2** | **Gene2_p** | **WC-related phenotype** |
| --- | --- | --- | --- | --- | --- | --- | --- | --- | --- | --- | --- | --- | --- | --- | --- | --- |
| rs1512847 | 1 | 210779343 | rs8022565 | 14 | 19866718 | < 1.00E-06 | < 0.05E-02 | 5.40E-01 | -1.68 | 2.20E-01 | 1.75 | *ATF3* | 6.03E-02 | *TTC5* | 3.70E-02 | men |
| rs12204218 | 6 | 5458549 | rs3807373 | 7 | 150299654 | < 1.00E-06 | < 0.05E-02 | 4.50E-01 | -1.55 | 3.60E-02 | -1.55 | *FARS2* | 1.94E-01 | *NOS3* | 3.54E-02 | women |
| rs2108794 | 7 | 156140469 | rs17335035 | 17 | 41185329 | < 1.00E-06 | < 0.05E-02 | 4.40E-01 | -1.78 | 3.90E-01 | -1.92 | *RNF32* | 1.60E-02 | *CRHR1* | 4.72E-03 | women |
