# Supplementary material for: Evidence of Polygenic Adaptation in the Systems Genetics of Anthropometric Traits
Source: PLoS One. 2016 Aug 18;11(8):e0160654. doi: 10.1371/journal.pone.0160654 (PMC4990182; doi:10.1371/journal.pone.0160654)
Supplement: S9 Table — NI: not included in top-10 PPI modules. (DOCX) [file pone.0160654.s009.docx]

**S9 Table**: Genes and their correspondence p values present in gene network associated with WHR-related phenotypes. NI: not included in top-10 PPI modules.

| **Gene** | **Distribution** | **Extreme phenotype differences** | **WHR-men** | **WHR-women** |
| --- | --- | --- | --- | --- |
| *ABI3* | NI | 8.40E-01 | NI | NI |
| *ACADVL* | NI | 8.40E-05 | NI | NI |
| *ACTBL2* | NI | 2.48E-02 | NI | 2.91E-03 |
| *ACTG1* | NI | 4.38E-02 | NI | NI |
| *ADA* | NI | NI | 7.04E-03 | NI |
| *AFP* | NI | NI | NI | 2.92E-01 |
| *AKT1* | 4.42E-02 | NI | 5.58E-04 | NI |
| *ANKFY1* | NI | 3.66E-04 | NI | NI |
| *ANTXR1* | NI | NI | 1.30E-03 | NI |
| *AP1G2* | NI | NI | NI | 3.76E-02 |
| *AP1S1* | NI | NI | NI | 5.27E-01 |
| *APBA1* | NI | NI | 5.66E-04 | NI |
| *APP* | 5.76E-02 | 3.69E-01 | 1.86E-01 | 1.06E-01 |
| *ARF5* | 4.84E-01 | NI | NI | NI |
| *ARHGEF11* | 7.02E-01 | NI | NI | NI |
| *ARHGEF2* | NI | 6.71E-03 | NI | NI |
| *ARMC9* | NI | 1.33E-01 | NI | NI |
| *ARPC2* | 1.74E-03 | NI | NI | NI |
| *ASGR1* | NI | 4.80E-05 | NI | NI |
| *BAP1* | 1.00E-06 | NI | NI | 1.59E-04 |
| *BCAS2* | NI | NI | NI | 4.05E-03 |
| *BCAT2* | NI | 1.40E-04 | NI | NI |
| *BEGAIN* | 1.61E-02 | NI | NI | 1.49E-02 |
| *C11orf68* | NI | NI | NI | 5.99E-01 |
| *C1orf105* | < 1.00E-06 | NI | < 1.00E-06 | 2.07E-04 |
| *CACNA2D3* | 4.62E-01 | NI | NI | NI |
| *CACNG2* | NI | NI | NI | 9.36E-01 |
| *CAMK1* | NI | 1.37E-02 | 2.68E-04 | NI |
| *CAP1* | 6.22E-01 | 2.75E-01 | NI | NI |
| *CAPNS1* | NI | NI | 5.87E-03 | NI |
| *CASP7* | 1.87E-01 | NI | NI | NI |
| *CASP9* | NI | NI | 7.82E-03 | NI |
| *CBL* | 1.48E-03 | NI | NI | NI |
| *CBR4* | NI | NI | 1.69E-01 | NI |
| *CCDC33* | 9.12E-03 | NI | NI | NI |
| *CCDC43* | NI | NI | 2.76E-02 | NI |
| *CD34* | NI | 1.04E-03 | NI | NI |
| *CDC25A* | NI | NI | 3.64E-02 | NI |
| *CDKAL1* | NI | 6.18E-04 | NI | NI |
| *CEBPA* | 2.98E-04 | NI | 8.46E-04 | NI |
| *CEP250* | NI | NI | 4.59E-03 | NI |
| *CEP72* | NI | NI | NI | 8.34E-03 |
| *CFB* | 4.79E-04 | NI | NI | NI |
| *CHEK2* | 3.07E-04 | NI | NI | NI |
| *CIB1* | NI | NI | NI | 1.18E-02 |
| *CKB* | NI | NI | 3.01E-02 | NI |
| *CLDN11* | NI | NI | NI | 7.40E-01 |
| *CLDN3* | 3.02E-01 | NI | NI | NI |
| *CLDN5* | 8.79E-01 | NI | NI | NI |
| *CLDN7* | NI | 6.40E-05 | NI | NI |
| *CLDN8* | NI | 5.19E-01 | NI | NI |
| *CLGN* | NI | 4.31E-03 | NI | NI |
| *CLK2* | NI | 2.23E-02 | NI | NI |
| *CMYA5* | NI | 8.29E-02 | NI | NI |
| *CNKSR1* | NI | NI | NI | 1.30E-02 |
| *CNTN1* | NI | 8.80E-01 | NI | NI |
| *COBLL1* | NI | NI | NI | < 1.00E-06 |
| *COL6A1* | 2.09E-02 | NI | NI | NI |
| *CORO7* | 5.00E-06 | NI | NI | 4.00E-05 |
| *CRTAP* | NI | 4.15E-01 | NI | NI |
| *CSDE1* | 1.10E-02 | NI | NI | 2.55E-03 |
| *CSNK1A1* | NI | 2.91E-02 | NI | NI |
| *CSNK2B* | 4.56E-03 | 1.08E-02 | NI | 5.41E-02 |
| *CSRP1* | NI | NI | NI | 2.27E-04 |
| *DAPK1* | NI | NI | 9.36E-03 | NI |
| *DAXX* | NI | NI | NI | 8.11E-03 |
| *DBI* | NI | 5.44E-02 | NI | NI |
| *DGCR14* | NI | 1.66E-03 | NI | NI |
| *DLG1* | NI | NI | NI | 5.28E-02 |
| *DLG4* | 8.78E-02 | 8.30E-05 | NI | NI |
| *DNAJA3* | 3.00E-05 | 9.41E-04 | NI | 1.18E-04 |
| *DNAJC28* | NI | NI | 6.02E-01 | NI |
| *DOT1L* | NI | NI | 1.17E-03 | NI |
| *DPPA3* | 2.95E-01 | NI | NI | NI |
| *DR1* | NI | NI | 1.61E-03 | NI |
| *DUSP6* | 5.84E-01 | NI | NI | NI |
| *DVL2* | NI | 7.10E-05 | NI | NI |
| *E2F3* | NI | 1.85E-03 | NI | NI |
| *EHD1* | 7.10E-05 | NI | 1.52E-04 | NI |
| *EHMT2* | 1.34E-03 | NI | NI | NI |
| *EIF5A* | NI | 1.34E-03 | NI | NI |
| *EIF6* | 2.30E-05 | NI | 1.80E-05 | NI |
| *ELAVL1* | NI | NI | 1.29E-02 | NI |
| *ENAH* | NI | 1.22E-02 | NI | NI |
| *EPRS* | 3.07E-03 | NI | NI | NI |
| *ERBB2* | NI | NI | 3.71E-02 | NI |
| *ERBB2IP* | 3.77E-03 | NI | NI | NI |
| *ERGIC3* | NI | NI | 8.93E-03 | NI |
| *ESR1* | NI | NI | 2.18E-02 | NI |
| *EYA1* | NI | NI | NI | 7.60E-01 |
| *F2RL2* | 4.22E-01 | NI | NI | NI |
| *FAF1* | NI | 3.08E-02 | NI | NI |
| *FAM83C* | NI | NI | 2.10E-05 | NI |
| *FBXL4* | NI | 7.88E-01 | NI | NI |
| *FBXO6* | NI | 5.68E-01 | NI | NI |
| *FBXW10* | NI | NI | NI | 3.64E-01 |
| *FCGR2C* | 8.46E-01 | NI | NI | NI |
| *FEZ1* | NI | NI | NI | 2.25E-03 |
| *FN1* | 8.59E-01 | NI | NI | NI |
| *FNBP1* | 8.14E-03 | NI | NI | NI |
| *FYN* | NI | 1.52E-01 | NI | NI |
| *GABARAP* | NI | 7.30E-05 | NI | NI |
| *GFI1* | NI | NI | NI | 1.21E-02 |
| *GGA1* | 3.05E-01 | NI | NI | NI |
| *GIPC1* | NI | NI | NI | 1.29E-02 |
| *GJA3* | NI | 3.05E-02 | NI | NI |
| *GLIS2* | NI | NI | NI | 2.32E-04 |
| *GMNN* | 3.79E-02 | NI | NI | NI |
| *GNAI2* | NI | NI | NI | 7.92E-02 |
| *GNB2* | NI | 7.42E-01 | NI | NI |
| *GNB5* | NI | 2.04E-01 | NI | NI |
| *GPS2* | NI | 1.44E-03 | NI | NI |
| *GPSM2* | NI | NI | NI | 2.17E-03 |
| *GRIK2* | 9.47E-01 | NI | NI | NI |
| *GTF3C2* | 4.21E-04 | 1.39E-03 | NI | NI |
| *HADH* | NI | NI | 7.42E-02 | NI |
| *HDAC1* | NI | 4.39E-01 | NI | NI |
| *HEY2* | NI | 1.09E-03 | NI | NI |
| *HGD* | NI | 5.86E-02 | NI | NI |
| *HINT2* | NI | NI | 4.15E-01 | NI |
| *HIVEP1* | NI | 9.40E-01 | NI | NI |
| *HJURP* | NI | NI | 1.73E-03 | NI |
| *HLA-B* | NI | NI | NI | 1.88E-01 |
| *HMGA1* | 3.62E-03 | NI | NI | 2.78E-04 |
| *HMGN2* | NI | 4.96E-03 | NI | NI |
| *HMOX1* | 1.65E-03 | NI | NI | NI |
| *HMOX2* | NI | NI | 1.32E-01 | NI |
| *HNRNPA0* | NI | NI | 5.80E-02 | NI |
| *HNRNPK* | NI | 3.51E-02 | NI | NI |
| *HOXA10* | 1.49E-03 | NI | NI | NI |
| *HOXA11* | 1.48E-03 | NI | NI | NI |
| *HOXA3* | NI | 1.55E-02 | NI | NI |
| *HOXC10* | 4.39E-04 | NI | NI | NI |
| *HOXC11* | 3.94E-04 | NI | NI | NI |
| *HPD* | NI | 5.05E-01 | NI | NI |
| *HSP90AA1* | NI | 9.80E-01 | NI | NI |
| *HSPA1A* | 3.07E-03 | 3.41E-02 | NI | NI |
| *HSPG2* | NI | NI | NI | 2.45E-01 |
| *HTATIP2* | 3.82E-01 | NI | NI | NI |
| *IKBKAP* | NI | NI | NI | 6.08E-03 |
| *IKBKE* | NI | NI | NI | 8.93E-02 |
| *IL7R* | NI | 1.35E-02 | NI | NI |
| *IMMT* | NI | NI | 1.99E-02 | NI |
| *IMPAD1* | NI | NI | NI | 5.97E-01 |
| *IMPDH1* | 3.51E-02 | NI | NI | NI |
| *IPO5* | 3.97E-01 | NI | NI | NI |
| *ITCH* | NI | 1.86E-03 | NI | NI |
| *ITGA2* | NI | NI | NI | 7.07E-02 |
| *ITIH4* | 3.66E-04 | NI | NI | NI |
| *ITPR1* | NI | NI | 3.77E-03 | NI |
| *JUN* | 9.36E-01 | NI | NI | NI |
| *KCND2* | 3.17E-02 | NI | NI | NI |
| *KCNE3* | NI | NI | NI | 4.52E-01 |
| *KDELR1* | NI | 9.80E-01 | NI | NI |
| *KIAA0408* | NI | NI | NI | 4.74E-03 |
| *KIAA1598* | NI | 5.59E-04 | NI | NI |
| *KIFC1* | 1.50E-01 | NI | NI | NI |
| *KIFC2* | 1.34E-01 | NI | NI | NI |
| *KISS1* | NI | NI | 1.13E-01 | NI |
| *KRT7* | NI | 7.95E-01 | NI | NI |
| *KRT82* | 7.46E-01 | NI | NI | NI |
| *LAMB1* | 4.06E-02 | NI | NI | NI |
| *LIMS1* | 3.34E-01 | NI | NI | NI |
| *LRP11* | NI | 5.40E-01 | NI | NI |
| *LUZP1* | NI | NI | NI | 3.22E-03 |
| *MAPK8IP2* | 1.48E-01 | NI | NI | NI |
| *MAPT* | NI | 4.11E-04 | NI | 1.33E-03 |
| *MCM5* | 1.35E-03 | NI | NI | NI |
| *MCRS1* | 1.81E-03 | NI | NI | 1.22E-02 |
| *MDFI* | 1.70E-02 | NI | NI | NI |
| *MDM2* | 2.13E-01 | NI | NI | NI |
| *MED9* | NI | NI | NI | 7.86E-03 |
| *MEIS1* | 2.21E-03 | NI | NI | NI |
| *MEN1* | 1.48E-04 | NI | 4.19E-04 | NI |
| *MINA* | NI | NI | NI | 4.24E-01 |
| *MMP24* | NI | NI | 3.00E-06 | NI |
| *MRPS6* | NI | 1.26E-04 | NI | NI |
| *MSH2* | NI | NI | 5.38E-02 | NI |
| *MTNR1A* | NI | NI | 8.72E-01 | NI |
| *MYH2* | 3.45E-01 | NI | NI | NI |
| *MYH9* | NI | NI | NI | 7.13E-03 |
| *MYPN* | 2.29E-01 | NI | NI | NI |
| *NCSTN* | NI | NI | NI | 2.41E-02 |
| *NDUFS5* | 9.58E-01 | NI | NI | NI |
| *NFATC1* | NI | NI | NI | 4.00E-06 |
| *NFATC2* | NI | NI | 7.99E-03 | NI |
| *NISCH* | 3.00E-06 | NI | NI | 3.60E-05 |
| *NMD3* | 3.78E-01 | NI | NI | NI |
| *NMT2* | NI | 3.25E-03 | NI | NI |
| *NPLOC4* | NI | 4.33E-02 | NI | NI |
| *NR2C2* | NI | 1.59E-04 | NI | NI |
| *NR3C2* | NI | 7.04E-01 | NI | NI |
| *NR4A2* | 5.39E-01 | NI | NI | NI |
| *NRD1* | NI | NI | NI | 6.30E-01 |
| *NRF1* | NI | 9.17E-02 | NI | NI |
| *NSF* | 1.82E-02 | NI | NI | NI |
| *NUDT22* | NI | NI | NI | 3.30E-01 |
| *OBFC1* | NI | NI | NI | 1.02E-02 |
| *OGG1* | NI | NI | 2.26E-04 | NI |
| *OPTN* | NI | 2.08E-01 | NI | NI |
| *OTUD6B* | NI | NI | 2.14E-04 | 8.78E-03 |
| *OVOL1* | NI | 7.69E-01 | NI | NI |
| *OXSM* | NI | NI | NI | 9.47E-01 |
| *PABPC1* | NI | 2.19E-02 | NI | NI |
| *PARP1* | 2.88E-03 | NI | 7.66E-03 | NI |
| *PARP12* | 5.43E-02 | NI | NI | NI |
| *PDE12* | NI | 5.59E-03 | NI | NI |
| *PDLIM5* | NI | 1.75E-02 | NI | NI |
| *PFAS* | NI | 2.83E-03 | NI | NI |
| *PFDN1* | NI | 1.39E-03 | NI | NI |
| *PHF23* | NI | 8.50E-05 | NI | NI |
| *PHF7* | < 1.00E-06 | NI | NI | 2.10E-05 |
| *PHGDH* | 2.43E-01 | NI | NI | NI |
| *PHLDA3* | NI | NI | NI | 3.13E-04 |
| *PICK1* | NI | NI | 5.86E-01 | NI |
| *PIK3R2* | 8.03E-01 | NI | NI | NI |
| *PLCG1* | 1.56E-02 | NI | NI | NI |
| *PLXND1* | NI | NI | NI | 4.00E-06 |
| *POLL* | NI | NI | 7.89E-01 | NI |
| *POLR2A* | NI | NI | NI | 6.32E-03 |
| *POU2F3* | 1.67E-01 | NI | NI | NI |
| *PPARG* | NI | NI | NI | 1.59E-04 |
| *PPM1G* | 4.22E-04 | 1.42E-03 | NI | 4.23E-04 |
| *PPP1R10* | NI | 2.21E-02 | NI | NI |
| *PPP1R14C* | NI | 1.94E-02 | NI | NI |
| *PPP1R9A* | NI | 9.19E-01 | NI | NI |
| *PRKAB2* | 1.10E-02 | NI | NI | NI |
| *PRKACB* | NI | 1.22E-03 | NI | NI |
| *PRKCD* | NI | 5.12E-03 | NI | NI |
| *PRKDC* | 8.98E-03 | NI | 1.14E-02 | NI |
| *PRPF4* | NI | 1.29E-02 | NI | NI |
| *PSENEN* | NI | NI | NI | 4.38E-01 |
| *PSG2* | NI | 7.90E-01 | NI | NI |
| *PSMB2* | NI | 2.12E-02 | NI | NI |
| *PTBP1* | NI | 2.64E-02 | NI | NI |
| *PTER* | 1.07E-03 | NI | NI | NI |
| *PTPN12* | 5.73E-03 | NI | NI | 1.75E-03 |
| *PURA* | NI | 3.34E-03 | NI | NI |
| *PWP1* | NI | 4.45E-01 | NI | NI |
| *PYGM* | 3.33E-04 | NI | NI | NI |
| *RAB1A* | NI | 2.69E-02 | NI | NI |
| *RAB3D* | 5.28E-01 | NI | NI | NI |
| *RAB3IP* | 9.36E-03 | NI | NI | NI |
| *RAD51C* | 1.77E-01 | NI | NI | NI |
| *RAD54L2* | NI | NI | NI | 4.81E-04 |
| *RAI1* | 2.07E-04 | NI | NI | 6.10E-05 |
| *RANGRF* | NI | 2.36E-03 | NI | NI |
| *RASD1* | NI | NI | NI | 8.04E-03 |
| *RASSF1* | NI | NI | NI | 6.00E-03 |
| *RBBP5* | NI | NI | NI | 9.62E-04 |
| *RCOR1* | NI | NI | NI | 4.32E-01 |
| *REV1* | NI | NI | 1.37E-01 | NI |
| *RGS19* | NI | NI | NI | 2.01E-02 |
| *RGS9* | NI | 9.11E-01 | NI | NI |
| *RIBC2* | NI | NI | NI | 6.84E-03 |
| *RNF150* | NI | NI | 2.02E-03 | NI |
| *RPA1* | 3.56E-02 | NI | NI | NI |
| *RPA3* | NI | 9.75E-01 | NI | NI |
| *RPL17* | NI | NI | 2.68E-03 | NI |
| *RPL18* | NI | 7.84E-03 | NI | NI |
| *RPL18A* | NI | 9.35E-01 | NI | NI |
| *RPL31* | NI | NI | NI | 8.97E-03 |
| *RPL5* | NI | NI | NI | 6.19E-03 |
| *RPS6KA1* | 4.71E-03 | 1.03E-02 | NI | NI |
| *RPS6KA5* | 4.18E-03 | 2.18E-03 | NI | 2.57E-03 |
| *SDHA* | NI | NI | NI | 7.28E-01 |
| *SEC23A* | NI | 8.26E-02 | NI | NI |
| *SF1* | 1.36E-04 | NI | 6.84E-04 | NI |
| *SF3A2* | 1.46E-03 | NI | 2.77E-03 | NI |
| *SLC2A4* | 1.12E-01 | 1.62E-04 | NI | NI |
| *SLC30A9* | NI | NI | 2.31E-01 | NI |
| *SLC5A6* | 1.03E-02 | NI | NI | NI |
| *SMURF1* | NI | 2.00E-01 | NI | NI |
| *SNAP25* | NI | NI | 9.68E-01 | NI |
| *SNRPB* | NI | NI | NI | 3.34E-03 |
| *SNUPN* | NI | NI | 1.93E-03 | NI |
| *SNX17* | 4.28E-04 | 1.34E-03 | NI | NI |
| *SOCS6* | NI | NI | NI | 1.66E-03 |
| *SREBF1* | NI | NI | NI | 9.60E-05 |
| *STAB1* | 1.00E-06 | NI | NI | NI |
| *STAT1* | NI | NI | 1.08E-02 | NI |
| *STK38L* | NI | 3.87E-04 | NI | NI |
| *TBX1* | NI | NI | NI | 7.18E-01 |
| *TERF2* | NI | NI | NI | 7.27E-01 |
| *TGM2* | NI | 1.71E-01 | 3.24E-03 | NI |
| *TIAL1* | NI | 7.68E-03 | NI | NI |
| *TINF2* | NI | NI | 7.35E-01 | NI |
| *TIPRL* | NI | 1.36E-02 | NI | NI |
| *TJP1* | NI | 4.01E-01 | NI | NI |
| *TMCC1* | NI | NI | NI | < 1.00E-06 |
| *TMOD2* | NI | NI | 4.76E-01 | NI |
| *TMPO* | NI | 9.67E-03 | NI | NI |
| *TNNC1* | 3.00E-06 | NI | NI | 4.70E-05 |
| *TOM1* | 2.40E-04 | NI | NI | NI |
| *TOM1L2* | NI | NI | NI | 1.07E-04 |
| *TOMM40* | NI | 5.60E-03 | NI | NI |
| *TP53BP2* | 1.95E-03 | NI | NI | 4.11E-04 |
| *TPM3* | NI | NI | 1.66E-01 | NI |
| *TRAF6* | NI | 6.07E-02 | NI | NI |
| *TRIM24* | 7.00E-02 | NI | NI | NI |
| *TRIM54* | 4.04E-04 | NI | NI | NI |
| *TSPAN3* | NI | NI | NI | 2.17E-01 |
| *TSPO* | NI | 2.36E-01 | NI | NI |
| *TTC23* | 9.60E-01 | NI | NI | NI |
| *TTN* | 9.00E-02 | NI | NI | NI |
| *TTR* | NI | NI | NI | 4.55E-01 |
| *TUBB* | NI | 6.18E-02 | NI | NI |
| *TUBB1* | 7.93E-01 | NI | NI | NI |
| *TYRP1* | NI | NI | NI | 5.91E-03 |
| *UBC* | 9.61E-01 | 7.76E-01 | 2.97E-01 | 9.38E-01 |
| *UBE2G1* | NI | 1.20E-03 | NI | NI |
| *UBE2I* | NI | NI | NI | 2.27E-02 |
| *UBL7* | NI | 3.35E-01 | NI | NI |
| *USP15* | 4.74E-02 | 5.00E-05 | NI | NI |
| *VAMP8* | NI | NI | 3.27E-02 | NI |
| *VASN* | 1.10E-05 | NI | NI | 5.20E-05 |
| *WARS2* | 4.60E-05 | NI | NI | NI |
| *WNK1* | NI | NI | NI | 9.11E-01 |
| *WT1* | 8.24E-04 | NI | NI | NI |
| *XBP1* | 8.00E-06 | NI | NI | NI |
| *XPNPEP1* | NI | 8.26E-03 | NI | NI |
| *XPO1* | 9.25E-02 | NI | NI | NI |
| *YWHAG* | 1.76E-01 | NI | NI | NI |
| *YWHAQ* | NI | NI | 5.44E-02 | NI |
| *YY1AP1* | NI | 9.04E-04 | NI | NI |
| *ZBTB9* | NI | NI | 6.82E-01 | NI |
| *ZC3H4* | NI | NI | NI | 3.36E-02 |
| *ZFP28* | NI | NI | 6.95E-01 | NI |
| *ZNF45* | NI | NI | 1.10E-02 | NI |
| *ZNF593* | 1.10E-04 | 4.00E-03 | NI | NI |
| *ZNHIT6* | NI | NI | 1.74E-01 | NI |
| *ZYX* | NI | 3.94E-02 | NI | NI |
