# Supplementary material for: Evidence of Polygenic Adaptation in the Systems Genetics of Anthropometric Traits
Source: PLoS One. 2016 Aug 18;11(8):e0160654. doi: 10.1371/journal.pone.0160654 (PMC4990182; doi:10.1371/journal.pone.0160654)
Supplement: S10 Table — (DOCX) [file pone.0160654.s010.docx]

**S10 Table**: Significant long-distance genotypic LDs observed in WHR-associated gene networks.

| **rsId1** | **chr1** | **loc1** | **rsId2** | **chr2** | **loc2** | **p.value** | **q.value** | **SNP1_p** | **SNP1_iHS** | **SNP2_p** | **SNP2_iHS** | **Gene1** | **Gene1_p** | **Gene2** | **Gene2_p** | **WHR-related phenotype** |
| --- | --- | --- | --- | --- | --- | --- | --- | --- | --- | --- | --- | --- | --- | --- | --- | --- |
| rs13077495 | 3 | 12302751 | rs2275635 | 9 | 110728588 | < 1.00E-06 | < 4.43E-08 | 1.40E-01 | -1.71 | 1.30E-01 | 1.55 | *PPARG* | 1.59E-04 | *IKBKAP* | *6.08E-03* | women |
| rs616788 | 3 | 54614249 | rs3131382 | 6 | 31815709 | < 1.00E-06 | < 4.43E-02 | 5.70E-01 | 1.53 | 3.10E-02 | -2.46 | *CACNA2D3* | 4.62E-01 | *BAT5* | *2.57E-03* | Distribution |
| rs358031 | 3 | 55027914 | rs6500605 | 16 | 4442411 | < 1.00E-06 | < 4.43E-02 | 6.40E-01 | 2.12 | 7.10E-04 | 2.10 | *CACNA2D3* | 4.62E-01 | *CORO7* | *5.00E-06* | Distribution |
| rs11720353 | 3 | 121911311 | rs1012635 | 6 | 20783274 | < 1.00E-06 | < 4.43E-03 | 6.00E-02 | 2.24 | 1.10E-01 | -1.61 | *HGD* | 5.86E-02 | *CDKAL1* | *6.18E-04* | extreme phenotype differences |
| rs10016497 | 4 | 141433672 | rs9509528 | 13 | 20565388 | < 1.00E-06 | < 4.43E-09 | 5.50E-01 | -1.97 | 4.60E-01 | 2.52 | *SCOC* | 5.05E-03 | *SAP18* | *2.67E-01* | extreme phenotype differences |
| rs1039987 | 4 | 141482111 | rs12246119 | 10 | 121317999 | 2.00E-06 | 4.43E-02 | 1.00E-01 | 2.75 | 3.40E-01 | 1.96 | *CLGN* | 4.31E-03 | *TIAL1* | *7.68E-03* | extreme phenotype differences |
| rs9266380 | 6 | 31442456 | rs2694927 | 17 | 17319229 | < 1.00E-06 | < 4.43E-04 | 3.90E-01 | -2.47 | 1.60E-02 | 1.92 | *HLA-B* | 1.88E-01 | *MED9* | *7.86E-03* | women |
| rs2844538 | 6 | 31456858 | rs5750250 | 22 | 35038429 | < 1.00E-06 | < 4.43E-06 | 1.40E-01 | 1.79 | 3.40E-02 | 2.36 | *HLA-B* | 1.88E-01 | *APOL1* | *5.58E-03* | women |
| rs9266773 | 6 | 31460327 | rs2694927 | 17 | 17319229 | < 1.00E-06 | < 4.43E-05 | 3.30E-01 | -3.48 | 1.60E-02 | 1.92 | *HLA-B* | 1.88E-01 | *MED9* | *7.86E-03* | women |
| rs296894 | 9 | 85788264 | rs2830113 | 21 | 26496353 | < 1.00E-06 | < 4.43E-07 | 1.10E-01 | -2.01 | 1.60E-01 | 2.38 | *HNRNPK* | 3.51E-02 | *APP* | *3.69E-01* | extreme phenotype differences |
| rs10734760 | 12 | 27239924 | rs10134356 | 14 | 90435400 | 2.00E-06 | 4.43E-02 | 2.30E-01 | 3.28 | 5.60E-01 | 1.98 | *STK38L* | 3.87E-04 | *RPS6KA5* | *2.18E-03* | extreme phenotype differences |
| rs6500605 | 16 | 4442411 | rs10485738 | 20 | 10100526 | < 1.00E-06 | < 4.43E-02 | 7.20E-02 | 2.10 | 5.50E-01 | 1.63 | *CORO7* | 3.33E-02 | *SNAP25* | *9.68E-01* | men |
